# Towards a comparative approach to the structure of animal personality variation

**DOI:** 10.1093/beheco/arz198

**Published:** 2019-11-26

**Authors:** Stephen John White, David John Pascall, Alastair James Wilson

**Affiliations:** 1 Animal Ecology, Department of Ecology and Genetics, Uppsala University, Uppsala, Sweden; 2 Institute of Biodiversity, Animal Health and Comparative Medicine, University of Glasgow, Glasgow, UK; 3 Centre for Ecology and Conservation, University of Exeter (Penryn Campus), Cornwall, UK

**Keywords:** animal personality, boldness, multivariate analysis, phylogeny

## Abstract

Latent personality traits underpinning observed behavioral variation have been studied in a great many species. However, a lack of standardized behavioral assays, coupled to a common reliance on inferring personality from a single, observed, behavioral trait makes it difficult to determine if, when, and how conclusions can be directly compared across taxa. Here, we estimate the among-individual (co)variance structure (**ID**) for a set of four behaviors expressed in an open field trial, putatively indicative of boldness, in seven species of small freshwater fish. We show that the **ID** matrices differ in terms of the total amount of variation present, and crucially the orientation, and as a consequence, biological interpretation of the first eigenvector. Specifically, loading of observed traits on the main axis of variation in **ID** matched a priori expectations for a shy-bold continuum in only three of the seven cases. Nonetheless, when the “shape” of the matrices was compared in higher dimensions, there was a high level of similarity among species, and weak evidence of phylogenetic signal. Our study highlights the present difficulty of trying to compare empirical inferences about specific personality traits across studies. However, it also shows how multivariate data collection and analysis allows the structure of behavioral variation to be quantitatively compared across populations or species without reliance on ambiguous verbal labels. This suggests that the field may have much to gain from greater uptake of phylogenetically informed comparative approaches when seeking to test evolutionary hypotheses about the origin and maintenance of personality variation.

## Teaser

A common animal personality test does not measure the same behavior in seven different species of tropical fish. The open field trial is often used to measure boldness, but by using a more thorough analysis we found that this test measured boldness in only three of the species and a stress response in the other four. Future work comparing personality among different species should ensure the reliability and efficacy of measurement methods used.

## INTRODUCTION

Animal personality is widely defined as the presence of consistent, among-individual behavioral variation that is stable over time. It is, therefore, a broad concept that encompasses a number of more specific axes of variation assumed to have generality across populations or species. The most well studied of these axes or “personality traits” include boldness, exploratory behavior, aggression, and sociality (Smith and Blumstein 2008; Bell et al. 2009; Toms et al. 2010). Personality traits can, therefore, be viewed as latent constructs that are not directly observed but can be studied from observed behaviors expressed during assays. If observable behaviors are repeatable for individuals, they can be used as proxies for underlying personality (Walsh and Cummins 1976; Carter et al. 2013; Araya-Ajoy and Dingemanse 2014).

Although personality traits have ostensibly been measured in a wide range of species, the design of experimental assays and the specific behavioral proxies measured typically differs between studies, making it difficult to assess the generality of findings. How can we be sure that multiple investigations of a personality trait are really studying the same biological phenomenon? Resolving this is challenging. For instance, the shy-bold continuum is arguably the most studied aspect of animal personality (Toms et al. 2010). Several assay types have been developed to capture this personality axis, including the emergence test, novel object tests and the open field trial, each with one or more behavioral proxies that could be observed (e.g., latency to leave a refuge in an emergence test). The variety of methods applied reflects legitimate disagreement over how to define—and so measure—particular personality axes. For instance, boldness is variously defined as the propensity to exhibit “risky” behaviors around novel objects, or as the behavioral response to a risky situation, not including response to novelty (Réale et al. 2007; Toms et al. 2010; Carter et al. 2013). It also reflects pragmatic considerations and logistical constraints arising from the biology of the study organism, whether assays are being conducted in the lab or field, and the need for relatively high throughput, repeated measures phenotyping (since data requirements for partitioning among-individual variation are high).

Thus, despite the abundance of empirical work, it is very difficult to compare inferences on personality variation across taxa, or even across studies of a single species. Here, we aim to address this gap, highlighting the utility of a more comparative approach to investigating personality variation. We do this using Open Field Trials (OFT) to characterize and compare the structure of personality variation across seven species of fish. The OFT is widely used to assay personality variation along a presumed “shy-bold” type continuum (Carter et al. 2012), although different researchers have also used it to infer latent personality traits of activity, anxiety, and exploration in birds (Dingemanse et al. 2002; Perals et al. 2017), mammals (Taylor et al. 2012), and fish (Sharma et al. 2009; Champagne et al. 2010; Adriaenssens and Johnsson 2013). Whether diverse conclusions reflect among-species differences in the structure of behavioral variation, and/or among-researcher differences in descriptive labels attached to that variation is unclear. However, we suggest that while continued debate over the latter is unlikely to contribute major biological insights, comparative studies to assess the former should be useful for evolutionary inference. For example, while adaptive explanations for personality variation dominate the literature, there has been little formal attempt to compare levels of variation among populations differing in (expected) selection regimes. We also know little of the extent to which phylogenetic signal is found in behavioral traits, and—in particular—whether more closely related populations or species have more similar patterns of among-individual (co)variation.

To adopt more formal comparative approaches in personality research, empiricists will need to apply common testing approaches to multiple species. However, this should also be coupled with fully multivariate data collection and analysis. The reasons for this are twofold. First, since latent personality axes are expected to be manifest in the expression of many observed behaviors (Walsh and Cummins 1976; Carter et al. 2013), it is intuitive that observing more behaviors should lead to their more robust inference. Considering multiple observed traits and their covariances together will give a more complete picture of the underlying latent variable(s) they are presumed to represent (Wright et al. 2006; Toms et al. 2010). Second, by using multivariate approaches and a common set of observed traits in a comparative context, we can employ quantitative comparison of the covariance structures among observed traits (Dochtermann and Roff 2010; Wilson et al. 2011; Brommer 2013; Houslay and Wilson 2017). This allows much more nuanced questions about the “shape” (as opposed to simply the amount) of among-individual behavioral variation and how it differs among populations/species. Despite these advantages, it remains true that most empirical studies of personality rely on univariate analyses of single behavioral proxies. Where researchers have sought to validate proxies by assessing correlation with other (plausible) measures of the same personality trait, the results have been mixed (Burns 2008; Toms et al. 2010; Perals et al. 2017). This highlights the simple point that two univariate studies of the same personality trait in the same population are likely to yield contrasting conclusions if they use different (but equally justifiable a priori) proxies.

In this study we aim to address the above argued need for comparative analyses of personality. We compare the among-individual behavioral variation structure of seven species of freshwater fish from three families Poeciliidae, Goodeidae, and Cyprinidae, taking a fully multivariate approach that utilizes repeated measures data from OFT to identify and compare among-individual axes of behavioral variation. The species used all inhabit freshwater streams where they may get swept to new and risky areas away from the shoal (Magurran 2005), thus an OFT (arguably) provides an ecologically relevant test of behavioral response to perceived risk in these species. We measure a common set of observed proxy traits—tracklength, activity, area covered and time in a middle zone across all species, and evaluate the extent to which the multivariate among-individual variation, estimated as an **ID** matrix, differs among species.

We compare **ID** matrices using several approaches to objectively quantify aspects of (dis)similarity. First, we compare the traces of **ID** to compare total amounts of among-individual variation. Second, we compare the leading eigenvector (principal component) of **ID** to ask whether the major axis of personality variation is similar across species. For instance, if observed traits are all valid proxies of a similar boldness axis, we would predict each species **ID** matrix will have a similar leading eigenvector along which all traits change in a positively correlated manner. In fact, we have already demonstrated this pattern is present in sheepshead swordtails (*Xiphophorus birchmanni*; Boulton et al. 2014) but not in guppies (*Poecilia reticulata*) (White et al. 2016). Thus our present aim is really to illustrate how multivariate methods allow simple verbal models about personality traits to be evaluated, rather than to test what we already know to be a “strawman” hypothesis. Third, we assess “subspace similarity” to determine if the matrices are similarly shaped when considering more dimensions than just the leading eigenvector. Finally, we test for phylogenetic conservatism in **ID** using a phylogenetic relatedness matrix between the species used that we construct from existing gene sequence data. All else being equal, we would expect more closely related species that share more of their evolutionary history, to have more similar behavioral traits than those more distantly related (as they do for other traits types; Uyeda et al. 2015). Whether this is true for the among-individual covariance structures that define personality (as opposed to the species-level average behaviors) is unclear and has not been considered previously to our knowledge.

## MATERIALS AND METHODS

### Study species and husbandry

We obtained repeated measures data from OFT of seven species of small freshwater fish from three families. These were the Trinidadian guppy (*Poecilia reticulata*), black-barred limia (*Lima nigrofasciata*), sheepshead swordtail (*Xiphophorus birchmanni*), green swordtail (*Xiphophorus hellerii*), and common platy (*Xiphophorus maculatus*) from the family Poeciliidae; the red-tailed splitfin (*Xenotoca eiseni*) from the family Goodeidae; and, the zebrafish (*Danio rerio*) from Capriniidae. All fish used were captive bred wild-type strains except for *X. maculatus* (which was an ornamental “blue tuxedo” strain). Data were collected at the University of Edinburgh’s Ashworth laboratories (*X. birchmanni and D. rerio*) and the University of Exeter, Penryn Campus fish facility (all other species) over various time periods between August 2010 and November 2016. Fish were kept at 21–25 °C (species dependent) on a 12:12 light–dark cycle and fed twice daily with commercial flake food and live brine shrimp nauplii (*Artemia salina*), frozen bloodworm or frozen adult brine shrimp (dependent on fish size). To allow individual recognition for repeated behavioral trials all fish used were tagged with either PIT tags implanted sub-dermally (*X. hellerii* using the P-Chip system at www.pharmaseq.com) or visible implant elastomer (all other species). All fish were tagged under anesthetic using a buffered MS222 solution as described elsewhere (White et al. 2016). The experiment was conducted under the auspices of the Animals (Scientific Procedures) Act 1986 under license from the UK Home Office (Project license number: P970C55C5) and with local ethical approval from the University of Exeter.

### Open field trials

Behavioral data collection was broadly similar across species, but with variation in numbers of fish (range of 26–831), average observations per fish (range 4–6) and experimental period (range 2–28 weeks; See Supplementary Table 1). Across all species, we obtained data from 5109 OFT on 1479 individuals. Data on *X. birchmanni* and *P. reticulata* have been published previously (Boulton et al. 2014; White et al. 2016). *Xiphophorus birchmanni* was unique in being assayed more times and over a longer period of 28 weeks as part of another study comparing short versus long-term measures of personality (Supplementary Table 1, Boulton et al. 2014). Data from other species have not previously been published.

The OFT procedure used has been detailed previously (Boulton et al. 2014; White and Wilson 2019) and we, therefore, abbreviate the current description. For all species, individual fish were assayed with multiple OFT, with at least 48 h between trials. Each OFT comprised an individual fish being transferred into a “bare” trial tank, lit from below using a lightbox and filled with 5 cm of water. For most species, the tank had a base of 45 × 25 cm, but for the smallest two (*D. rerio, P. reticulata*) we elected to use a smaller tank (30 × 20 cm base, Supplementary Table 1). Following a 30-s settling time, fish movement was tracked using an overhead camera and Viewer software (www.biobserve.com) over a 4 min 30 s time period for most species (trials lasted 5 min for *X. birchmanni* and *D. rerio*). Tracklength (Tl, defined as the total length (cm) that the individual swam), activity (Act, percent of the time spent moving over 4 cm/s) and area covered (AC, percentage of the tank area covered) were extracted from the tracking data. In addition, central and outer zones (of equal area; see Boulton et al. 2014) were imposed on the tank using Viewer software and the time spent in the middle zone (TIM, measured in seconds) was also recorded. The OFT water was not changed after each trial, but rather after each group of individuals (with different group sizes across species; Supplementary Table 1). Effects of order (within group) could arise from cumulative effect of netting stress from the home tank (groups corresponded to sets of fish housed together) and/or buildup of chemical cues in the OFT tank, so are controlled for statistically (see below).

### General statistical methods

As the species used varied in average size (smallest by standard length being *P. reticulata* at 19.47 mm and largest being *X. eiseni* at 48.25 mm), we decided to scale each individual tracklength measure by average species length to produce distance swam in (average) body lengths. We elected to do this, rather than dividing each individual’s TL by its own SL as the latter risks conflating personality with within-species size variation (i.e., our scaling retains any size-dependent among-individual variation within each species). For all species, TIM was square-root transformed to better fit the assumption of residual normality required for our linear mixed effect models (see below). All (transformed) traits were then mean-centered and scaled to standard deviation units (SDU). In doing this, we use the global (i.e., across all individuals of all species) mean and standard deviations. This puts all traits on a similar scale, aiding convergence of multivariate mixed models while still maintaining differences between species. See Supplementary Table 2 for mean values for scaled (globally across all species) data used in analyses, mean values of raw unscaled data, and TL means unscaled by SL (in cm rather than body lengths).

Data were primarily analyzed using linear mixed-effect models fitted in ASReml-R (Butler et al. 2009) using restricted maximum likelihood (REML). The four traits assayed are significantly repeatable in *P. reticulata* (White et al. 2016) and *X. birchmanni* (Boulton et al. 2014) but to get comparable estimates (and tests of) repeatabilities we first fitted univariate mixed models to each trait in each species. Each model included a random effect of individual identity (FishID). A fixed factor of repeat (cumulative number of trials experienced) and continuous linear effect of order within-group were also fitted. These were included to control for any across-trial habituation to the OFT and/or trends within groups respectively. Repeatability, conditional on these fixed effects, was estimated as the intraclass correlation coefficient (V_I_ /(V_I_+V_R_)) where V_I_ is the among-individual variation and V_R_ is the residual (within-individual) variance. The significance of V_I_ was determined by likelihood ratio test (LRT) comparison to a simpler model with no random effect of FishID. As a single random effect was tested, the test statistic was assumed to follow a 50:50 mix of χ ^2^ with 0 and 1 degrees of freedom (Visscher 2006).

### Among-species variation in mean behavioral phenotype

Before comparing the multivariate among-individual variance-covariance structures between the seven species, we first described differences in (average) multivariate phenotype. Canonical variate analysis (CVA) was used to do this, visualizing the spread of the species across multivariate trait space. Since all traits were repeatable (see results) and we wished only to describe qualitative patterns in the raw data, we elected—for simplicity—to calculate a within-individual mean behavior for each trait (rather than, for instance, reducing to a single multivariate mean phenotype per individual using mixed model predictions and their uncertainty; Araya-Ajoy & Dingemanse 2014). CVA was then applied as a data reduction approach (using the lda function from the MASS R package and ggplot2 from the tidyverse package) to identify and visualize the main, orthogonal axes of variation (canonical variates) across pre-specified groups, in this case, species. Note the distinction with PCA in which no a priori groups are defined. This is done by sequentially maximizing the differences between the groups in a similar fashion to principle component analysis (Campbell and Atchley 1981). This technique has been used to describe multivariate differences in both behavioral and morphological traits (Carter and Feeney 2012; Figueirido et al. 2016).

### Multivariate models to estimate species-specific **ID** matrices

For each species, we then estimated the among-individual behavioral (co)variance matrix, **ID,** for the set of four traits (Tl, Act, AC, and TIM) using multivariate mixed models. As with the univariate models above, fixed effects of repeat and order caught were fitted for each trait along with a random effect of FishID. This allowed us to estimate **ID** conditional on these fixed effects for each species. We tested for significant among-trait covariance structure in the **ID** matrix of each species by comparing the full model fit to a reduced model in which all covariance terms in **ID** were fixed at zero using a LRT on 6 DF.

We then tested for each pair of species, s1 and s2, the null hypothesis that **ID**_**s1**_ = **ID**_**s2**_. To do this, we fitted a series of eight-trait cross-species multivariate models (i.e., each of the four OFT traits for the first species fitted with the four traits for second) with fixed and random effects as described above (but with no cross-species covariance terms). Thus, for each species pair we estimate **ID**_**s1,s2**_ as a blocked matrix where

$$\begin{array}{l} ID_{S1,S2}=\left[\begin{array}{ll} ID_{S1} & 0\\ 0 & ID_{S2} \end{array}\right] \end{array}$$ (1)

The model fit was then compared (LRT at 10 DF) to a simplified model in which we impose the constraint that **ID**_**s1**_ = **ID**_**s2**_. We note that there are 21 species pairs in total and thus this analysis creates a multiple testing issue that is not easily resolved (as comparisons are nonindependent by design) so we stress that resulting *P* values are nominal.

### Among-species comparison of **ID**

While the above tests against a null hypothesis for equality of **ID** between species, a more nuanced approach is to describe in what way, and to what extent the matrices are (dis)similar. We chose three complementary approaches to this. First, we asked whether the species differed in the total amount of among-individual behavioral variance, calculated as the trace of **ID** (i.e., the sum of diagonal elements corresponding to V_I_ estimates for the four traits). For each species, approximate 95% CI of the trace were determined from a 5000 draw parametric bootstrap (following Boulton et al. 2014; White et al. 2019), and for each species pair we estimated the absolute value of the difference in traces, Δ _s1,s2_.

Second, we asked whether the leading eigenvectors of the among-individual covariance matrixes (denoted **ID**_**max**_) were similar among species. To do this, we calculated the angle θ between **ID**_**max(s1)**_ and **ID**_**max(s2)**_ for each species pair (s1, s2). Eigenvector decomposition is useful in determining the extent to which observed traits map to an underlying model or expectation of personality. Thus, for example, if all four observed traits represent valid proxies of a single latent personality trait (e.g., boldness), and that personality trait is structurally similar in two species then θ will be low (on a scale ranging from 0° when vectors are perfectly aligned to 90° if orthogonal).

Third, we used the Krzanowski subspace comparison test to obtain a general index of similarity between higher dimensional subspaces of the **ID** matrices (krzanowski.test function from the MCMCglmm package; Hadfield 2010). This is useful if **ID**_**max**_ does not account for a clear majority of variation. In such cases, two species may differ dramatically in one dimension (i.e., have poor alignment of their respective leading vector in trait space), but be very similar when compared across two (or more) dimensions. Take two *n*-dimensional covariance matrices (i.e., each describing covariance among a set of *n* traits). For matrix subspaces defined by a number of eigenvectors *x* (where *x* ≤ *n*/2) we can calculate the similarity index, K (Krzanowski 1979), by rotating the chosen eigenvectors to minimize the angle between them. Specifically, we first define the matrix **S** as:

$$\begin{array}{l} S\;=\;A^{T}BB^{T}A \end{array}$$

Where **A** and **B** contain the subset of *x* eigenvectors of the two original covariance matrices being compared, and superscript T denotes their transpose. The similarity index K is then equal to the sum of the eigen values of the **S matrix**, and has a lower limit of 0 (subspaces are maximimally unrelated) and an upper limit equal of *x* (meaning completely alignment in the chosen subspace dimensionality; (Krzanowski 1979; Blows and Walsh 2007; Aguirre et al. 2014). Here, we chose to compare **ID** matrices among species in two-dimensional subspace (see Results for justification of this choice), such that 0 ≤ K ≤ 2.

### Testing for phylogenetic signal in **ID**

Finally, since the matrix comparison tools described above yield three different measures of **ID** matrix dissimilarity, we asked whether these are predicted by phylogeny. The limited number of species (*n* = 7) precludes formal comparative analysis using, for instance, phylogenetic mixed model approaches. Consequently, we simply estimated the correlations between among-species matrices based on Δ, θ, and K and a phylogenetic distance matrix among species (determined as described below). Note that since Δ and θ are actually measures of dissimilarity and K increases with similarity, for consistency, we used a matrix containing pairwise (between species) values of 2-K. This means we predict all correlations will be positive if there is a phylogenetic signal (i.e., **ID** matrices become more dissimilar as phylogenetic distance between species increases).

While the phylogenetic relatedness between some of the species used here has been studied (Marcus and McCune 1999; Hamilton 2001; Jones et al. 2013), there is no published phylogeny including all of the seven species used here. For each *of the* seven species, all available sequences for six genes were obtained from Genbank (https://www.ncbi.nlm.nih.gov/genbank). The genes chosen were tyrosine kinase (XSRC), SH3PX3, rhodopsin, recombination activating gene 1 (RAG1), ectoderm-neural cortex protein 1 (ENC1), and cytochrome c oxidase subunit 1 (COI). Any sequences predicted from the genome (accession IDs starting with XM, XR, or XP), after the first believed to represent duplicate sequences from the same laboratory strain, and any sequences containing introns were discarded. See Supplementary Table 3 for the sequence IDs, associated metadata and references of included sequences.

The sequences were aligned using the L-INS-i mode in MAFFT with “maxiterate” set to 16 (Katoh et al. 2005). In all cases, the alignments were trimmed from the start (and end) until at least three sequences were present in the first (or last) column for computational reasons. Phylogenetic analysis was performed in BEAST2 v2.4.8 (Bouckaert et al. 2014). A species-level phylogeny with a Yule tree prior was estimated in StarBeast2 v0.14.0 using all sequences (Ogilvie et al. 2017). The ploidy for all genes except COI was set to 2, with the mitochondrial COI being set to 0.5. For all genes, bModelTest v1.0.4 was used to account for uncertainty in the underlying evolutionary model (Bouckaert and Drummond 2017). A strict clock was used for all sequences with the clock rate of XSRC being fixed at 1, and all other genes clock rates being estimated relative to this. Exponential (λ = 1) priors were placed over the gamma shape parameters of all genes. A weakly informative Gamma (α = 0.1, β = 1000) prior was placed over the speciation rate. Log-normal (μ (in real space) = 1, SD = 1) priors were placed over the clock rate relative to XSRC for each gene. All other priors were left at their default values. Four runs were run for 1 000 000 000 iterations with every 5000th tree being stored. We removed 50% for burn-in and thinned to 1000 independent trees. Convergence was assessed in Tracer v1.7 (Rambaut et al. 2017). The posterior distribution of trees was thinned to 1000 trees to ensure all trees represented independent draws from the posterior.

Phenetic distances were calculated between all species for every tree in the posterior distribution of trees using the function cophenetic in the ape package in R. The correlation between the evolutionary distances and the behavioral distances was estimated using Kendall’s tau-b (to account for the likely nonlinear relation of the two variables) from the mantel function in the R package vegan.

A bootstrap approach was used to account for the uncertainty introduced by the uncertainty in the inputs. A random evolutionary distance matrix was selected from the posterior distribution of matrices, then a series of random draws from the bootstrap distributions of the behavioral dissimilarity matrices were used to create a matching matrix of distances between species for each parameter. The correlation was then calculated. This was performed 1000 times, and the 95% confidence intervals were taken as 2.5th and 97.5th percentiles of the bootstrap distribution of the Kendall’s tau-b statistic from the mantel function output.

## RESULTS

**Figure 1:**
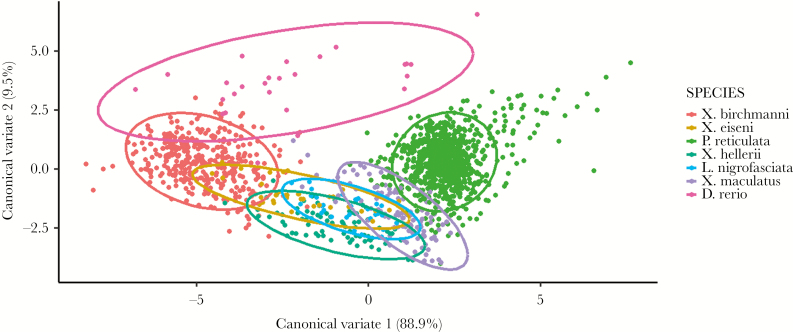
CVA of all 7 species, with individuals plotted on the first two canonical variates. Ellipses show approximate 95% confidence intervals for the phenotypic distributions of each species.

Our univariate mixed models showed all traits are significantly repeatable in all species. Repeatability estimates were low to moderate, ranging from 0.157 (Act in *D. rerio*) to 0.562 (TIM in *D. rerio*) with a median value of 0.366 across traits and species (all estimates presented in Supplementary Table 4). The CVA also shows clear separation of (average) behavioral phenotypes between some, but not all species (Figure 1). Thus based on the first two canonical variates, *X. hellerii, L. nigrofasciata, X. maculatus, and X. eiseni* appear quite similar to each other in behavioral phenotype, forming a “core group” of species with large overlap of confidence ellipses (Figure 1). *Xiphophorus birchmanni* and *P. reticulta* are moderately differentiated from this group, and more strongly from each other along CVA1. This axis captures most of the among-species variance (88.9%) and loads antagonistically on mean TL and mean Act (Coefficients of linear discriminants for within-individual mean behaviors: TL = 0.124, Act = −0.176, AC = 0.023, TIM = −0.033). Thus, for instance, relative to *X. birchmanni*, it is the case that *P. reticulata* tends to exhibit greater track lengths but less time active. *Danio rerio* is strongly differentiated from the core group as well as from *P. reticulata*, but there is some overlap of confidence ellipses with *X. birchmanni*. Differentiation of *D. rerio* is primarily on CVA2, which captures 9.5% of the among-species variance and loads primarily on TIM (Coefficients of linear discriminants for within-individual mean behaviors: TL = 0.034, Act = 0.025, AC = 0.026, TIM = 0.148). Thus separation of *D. rerio* is largely driven by an increased tendency of this species to spend time in the middle of the OFT arena. Interestingly this species also shows particularly high variation on CVA1.

Multivariate mixed models provided evidence of significant among-individual (co)variance structure in **ID** for all poeciliids and for the goodeid *X. eiseni* (LRT comparison of full and reduced models, all *P* < 0.001; Supplementary Table 5). However, *D. rerio* provided an exception to this pattern (LRT comparison, $\chi_{6}^{2}=10.46$, *P* = 0.107) and so we cannot exclude the possibility that among-trait covariance is entirely due to within-individual effects in this species. Nonetheless, our unconstrained (i.e., with covariance terms modeled) estimate of the *D. rerio***ID** matrix provides our best estimate of the among-individual variation and was used in all subsequent matrix comparisons.

### Among-species comparison of **ID** matrices

Estimates of **ID** obtained for all species (s) are shown in Supplementary Table 6. Pairwise testing for equality of **ID** using the eight-trait multivariate models proved somewhat problematic as, for reasons we have been unable to determine, we were unable to obtain stable model convergence when *L. nigrofasciata* was one of the species in the pair. However, for most species pairs we found evidence against the null hypothesis of equal **ID** matrices (at a nominal α = 0.05 with no correction for multiple testing; Supplementary Table 7). One exception to this was the comparison between *X. hellerii* and *X. eiseni* comparison ($\chi_{10}^{2}=5.41$, *P* = 0.862). We, therefore, conclude that there is some evidence of among species differences in **ID** matrices.

### Trace comparisons

**Figure 2:**
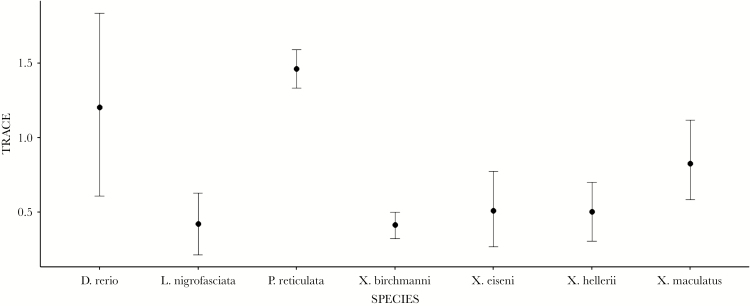
Total multivariate variance (trace) for each species. 95% CI shown.

Matrix trace comparisons provide further support for differences in among-individual (multivariate) behavioral variance (Figure 2). *Xiphophorus hellerii, L. nigrofasciata, X. eiseni*, and *X. birchmanni* have very similar traces (ranging from 0.415 to 0.513 standard deviation units (SDU)) and all estimates of Δ between these species are non-significant (based on approximate 95% CI overlapping zero; Table 1). However, the common platy *X. maculatus* has a slightly larger trace of 0.828 SDU, which is significantly greater than both *L. nigrofasciata* and *X. birchmanni* (Table 1). *Poecilia reticulata* is most dissimilar to the other species, having the highest trace (1.463 SDU) and showing significantly more among-individual variance than all species except *D. rerio*. Due to the large 95% CI surrounding the trace of *D. rerio*, it is difficult to comment on its trace similarity with the other species, but the point estimate of the trace is at least qualitatively more similar to *P. reticulata* than the other species. A closer examination of the **ID** matrix estimates (Supplementary Table 6) shows that increased amounts of variance in these two species are largely due to increase in V_I_ for TL and to a lesser extent in AC and TIM. Note that trait units of analysis here are global (i.e., across all observations on all species) SDUs and this pattern in V_I_ is not seen in the repeatabilities (which are standardized by total phenotypic variance within each species).

**Table 1:** Estimates of Δ, the absolute value of the difference in **ID** matrix traces (with 95% CI) between each species pair

	*Danio rerio*	*Lima nigrofasciata*	*Poecilia reticulata*	*Xiphophorus birchmanni*	*Xenotoca eiseni*	*Xiphophorus hellerii*
*L. nigrofasciata*	0.783 (0.147, 1.498)					
*P. reticulata*	0.258 (−0.893, 0.441)	1.042 (0.795, 1.28)				
*X. birchmanni*	0.790 (0.095, 1.405)	0.007 (−0.25, 0.207)	1.049 (0.882, 1.197)			
*X. eiseni*	0.692 (−0.016, 1.405)	0.091 (−0.235, 0.443)	0.951 (0.651, 1.232)	0.098 (−0.165, 0.368)		
*X. hellerii*	0.700 (0.021, 1.387)	0.084 (−0.213, 0.372)	0.958 (0.699, 1.174)	0.090 (−0.117, 0.309)	0.008 (−0.31, 0.325)	
*Xiphophorus maculatus*	0.377 (−0.299, 1.122)	0.406 (0.061, 0.739)	0.636 (0.327, 0.916)	0.413 (0.135, 0.715)	0.315 (−0.053, 0.682)	0.323 (−0.01, 0.66)

### Alignment of leading eigenvectors

**Figure 3:**
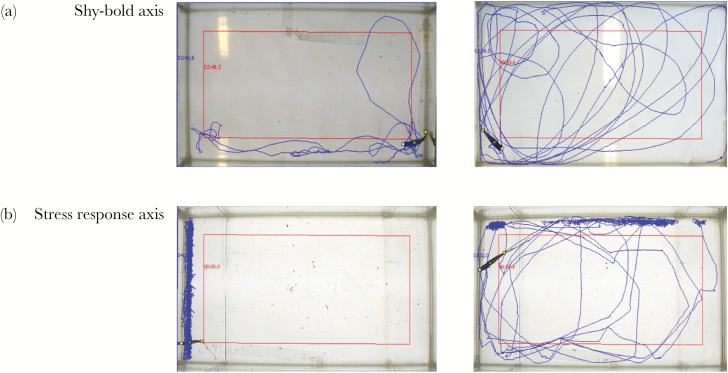
Example tracks to illustrate two of qualitative types of variation captured by IDmax interpreted as (a) a shy-bold personality continuum and (b) an axis of variation associated with stress response. Blue lines show the track of a fish in the arena during a 4.5-min OFT, whereas red lines distinguish an inner “middle zone” from an outer region of equal area close to the tank walls. In (a) a putatively shy track (left) is contrasted with a bold one (right). In (b) the left track depicts a “flight” type stress response characterized by very rapid swimming along one (in this case) or more walls of the arena, whereas the track on the right shows an individual that has spent less time engaging in this behavior and has been more exploratory. These screenshots were taken from the Viewer software. Examples in (a) are trials of *Lima nigrofasciata* and in (b) of *Xiphophorus hellerii*.

Eigenvector decomposition reveals **ID**_**max**_ accounts for an estimated minimum of 58% (*D. rerio*) and maximum of 85% (*X. maculatus*) of variance in **ID**, with a median of 60.4% across species. Based on how the four traits load on **ID**_**max**_ in each species, a qualitative—and admittedly subjective—interpretation is that the OFT is revealing three different types of personality variation (Table 2). In *X. birchmanni, L. nigrofasciata,* and *X. maculatus* the trait loadings match our *a priori* “shy-bold” model, with all OFT traits loading with the same sign on **ID**_**max**_ (Figure 3a). Biologically, this means that for these species, individuals with consistently longer *tracklengths* also have higher *activity* and *area covered*_,_ and spend more time in the middle of the arena. In contrast, for *X. hellerii* and *P. reticulata*, Tl and Act load on **ID**_**max**_ in a direction that is antagonistic to AC and TIM. We have argued elsewhere that, at least in relation to guppies, this pattern is consistent with variation in behavioral stress response (White et al. 2016, Houslay et al. 2018). Specifically, some individuals exhibit “flight” type behaviors, swimming rapidly one or more tank edges seeking an escape from the arena (leading to high Tl and Act but relatively low AC and TIM; see Figure 3b for an illustrative example). The third axis type, seen in *D. rerio* and *X. eiseni*, has TIM loading antagonistically to all other traits. This structure of variation is not easy to attach an intuitively descriptive label to, but clearly differs from our naive shy-bold paradigm because apparently bold individuals (as might be inferred from traits other than TIM) are more thigmotaxic not less. More quantitatively, estimates of θ indicate support for this grouping, with relatively low angles between **ID**_**max**_ of species of similar axis type (e.g., θ = 20.3° between *X. maculatus* and, *L. nigrofasciata*; θ = 31.6° between *X. hellerii* and *P. reticulata*), but poor alignment in other cases (e.g., θ = 89.2° between *X. birchmanni* and *X. hellerii* revealing the major axes to be effectively orthogonal) (Table 3).

**Table 2:** The first (a) and second eigenvector (b) of **ID** each species, with associated eigen values, percent of total among-individual variance explained and the loadings of each trait on the vectors

a)	*Danio rerio*	*L. nigrofasciatia*	*Poecilia reticulata*	*Xiphophorus birchmanni*	*Xenotoca eiseni*	*Xiphophorus hellerii*	*Xiphophorus maculatus*
Eigen value	0.704	0.261	0.842	0.250	0.348	0.305	0.706
Percentage	58.388	62.010	57.570	60.391	67.892	60.352	85.349
Loadings							
TL	0.760	0.165	−0.593	0.563	0.321	−0.192	0.237
Act	0.173	0.354	−0.436	0.583	0.728	−0.423	0.383
AC	0.469	0.853	0.353	0.495	0.310	0.134	0.644
TIM	−0.416	0.346	0.578	0.314	−0.521	0.875	0.619
b)							
Eigen value	0.368	0.149	0.407	0.139	0.156	0.178	0.112
Percentage	30.576	35.352	27.785	34.477	30.472	35.313	13.561
Loadings							
TL	0.192	0.233	0.541	0.376	0.099	0.304	0.333
Act	0.192	0.540	0.357	0.337	0.171	0.615	0.513
AC	0.395	0.057	0.614	−0.315	0.682	0.682	0.283
TIM	0.878	−0.806	0.450	−0.804	0.704	0.258	−0.739

**Table 3:** Angle θ between estimates of **ID**_**max**_ for each species pair (with approximate 95% CI in parentheses)

	*Danio rerio*	*Lima nigrofasciata*	*Poecilia reticulata*	*Xiphophorus birchmanni*	*Xenotoca eiseni*	*Xiphophorus hellerii*
*L. nigrofasciata*	63.7 (32.2, 90.0)					
*P. reticulata*	73.1 (6.5, 83.1)	75.6 (37.4, 89.9)				
*X. birchmanni*	50.9 (24.3, 89.3)	33.9 (22.7, 79.7)	76.6 (56.9, 89.9)			
*X. eiseni*	42.9 (31.9, 83.6)	66.8 (15.2, 89.8)	45.6 (26.1, 76.7)	53.5 (24.2, 86.4)		
*X. hellerii*	58.7 (32.9, 89.9)	76.4 (17.650, 89.9)	31.6 (24.7, 66.3)	89.2 (33.8, 89.9)	38.4 (4.7, 84.2)	
*Xiphophorus maculatus*	73.1 (26.0, 89.9)	20.3 (9.1, 74.2)	73.9 (62.4, 85.5)	29.6 (9.1, 51.5)	76.6 (34.7, 89.9)	65.1 (21.0, 89.9)

### Among-species similarity of **ID** in two-dimensional subspace

**Figure 4:**
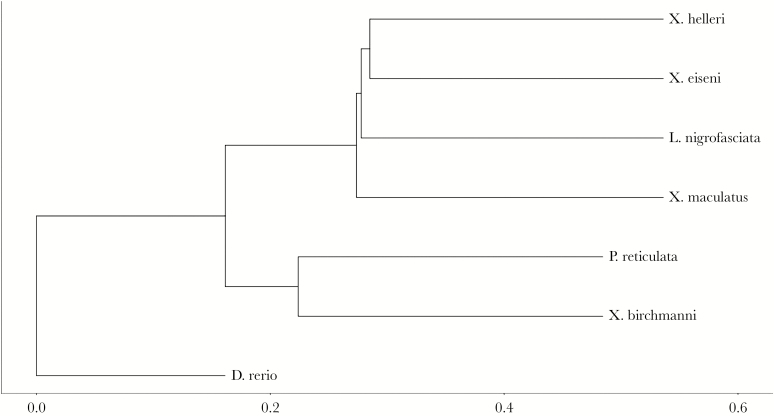
Representation of behavioral ID matrix “distances” between species in two-dimensional phenotypic subspace based on use of 2-K as a pairwise measure of dissimilarity.

As noted above, while **ID**_**max**_ captures approximately 85% of among-individual variance in *X. maculatus*, it was less dominant for the other species (in which the second eigenvector explained a relatively substantial 27.8% to 35.3% of total variance in **ID**; Table 2). Nonetheless, the first two eigenvectors together did capture the great majority (88–99%) of variation in all species (Table 2) justifying our decision to compare 2-dimensional subspaces between the matrices with the Krzanowski tests. Across species pair comparisons, 2-K ranged from 0.009 to 0.567 (on a scale from 0 to 2) indicating that, despite poor alignment of **ID**_**max**_ in many instances, the structure of variation is actually relatively similar in two-dimensional subspace (Table 4, Figure 4).

**Table 4:** Krzanowski’s index of two-dimensional subspace dissimilarity (2-K) among species-specific **ID** matrix estimates (with approximate 95% CI in parentheses)

	*Danio rerio*	*Lima nigrofasciata*	*Poecilia reticulata*	*Xiphophorus birchmanni*	*Xenotoca eiseni*	*Xiphophorus hellerii*
*L. nigrofasciata*	0.567 (0.279, 1.144)					
*P. reticulata*	0.452 (0.292, 1.084)	0.464 (0.296, 0.688)				
*X. birchmanni*	0.365 (0.216, 1.112)	0.319 (0.175, 0.535)	0.082 (0.040, 0.151)			
*X. eiseni*	0.547 (0.305, 1.232)	0.039 (0.001, 0.694)	0.372 (0.247, 0.722)	0.194 (0.098, 0.500)		
*X. hellerii*	0.482 (0.272, 1.114)	0.066 (0.001, 0.376)	0.379 (0.271, 0.541)	0.170 (0.085, 0.345)	0.009 (0.000, 0.367)	
*Xiphophorus maculatus*	0.324 (0.133, 1.043)	0.097 (0.007, 0.631)	0.440 (0.157, 0.764)	0.212 (0.032, 0.511)	0.067 (0.003, 0.653)	0.037 (0.002, 0.344)

### Evidence of phylogenetic signal in the structure of **ID**

**Figure 5:**
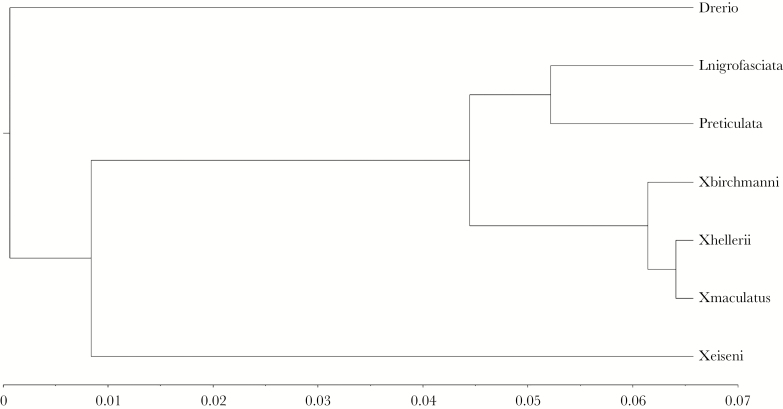
Phylogram of phylogenetic distance between the species based on sequence data for six genes obtained from Genbank.

The resultant phylogeny (Figure 5) is fully consistent with expectations from other studies (Marcus and McCune 1999; Hamilton 2001; Jones et al. 2013). Expressing this structure as a phylogenetic distance matrix (Table 5), we estimated correlations of *r* = 0.076 (95%CI lower = −0.187; 95% CI upper = 0.366) and *r* = −0.107 (95%CI lower = −0.207; 95% CI upper = 0.297) with dissimilarity matrices based on Δ and θ, respectively. However, a much stronger correlation of 0.320 (95%CI lower = 0.016; 95% CI upper = 0.570) was estimated between phylogenetic distance and subspace dissimilarity matrix (defined from pairwise values of 2-K). Thus phylogenetic distance between species appears to explain little among-species variation in trace or direction of the leading eigenvector. However, there is some evidence that it does explain differences between species in the overall “shape” of among-individual behavioral variation (i.e., personality variation) as characterized through OFT and captured by the two-dimensional subspace of **ID**.

**Table 5:** Phylogenetic distance between species, given as the difference in the number of nucleotide substitutions

	*Danio rerio*	*Lima nigrofasciata*	*Poecilia reticulata*	*Xiphophorus birchmanni*	*Xenotoca eiseni*	*Xiphophorus hellerii*
*L. nigrofasciata*	0.132					
*P. reticulata*	0.132	0.029				
*X. birchmanni*	0.132	0.045	0.045			
*X. eiseni*	0.132	0.129	0.129	0.129		
*X. hellerii*	0.132	0.045	0.045	0.012	0.129	
*Xiphophorus maculatus*	0.132	0.045	0.045	0.012	0.129	0.001

A larger value between two species indicates greater phylogenetic distance.

## DISCUSSION

Here we asked whether the OFT, a commonly used personality assay, revealed among-individual behavioral variation that was similarly structured across seven species of small fish. We also asked whether the variation characterized within each species was consistent with a priori expectations under a simple shy-bold paradigm, and whether— if differences in personality structure between species were apparent—they were predicted by phylogeny. Our results show that these superficially simple questions require somewhat nuanced answers. There are important differences between species in both the amount and structure of among-individual variation revealed by this standardized testing. For instance, **ID**_**max**_ was readily interpretable as a shy-bold axis in some but not all cases. This result was expected given previous characterization of **ID** in guppies and sheepshead swordtails (Boulton et al. 2014; White et al. 2016). However, it illustrates that—even under a common methodological approach in closely related taxa—variation labeled as “boldness” in one study can be structurally (and perhaps functionally) different from variation receiving the same label in another. This only becomes apparent in multivariate trait space, which is why assaying multiple behavioral proxies is important.

At the same time, and despite differences in orientation of the leading eigenvectors, all pairwise species comparisons of **ID** actually had moderate to high similarity as captured by the orientation of two-dimensional subspace. In other words, the structure of the “shape” of the behavioral variation revealed by OFT is only dramatically different between species when we focus just on a single dimension (i.e., assume all traits are assaying a single latent “personality” axis). We also find that estimated matrix similarity in two-dimensional subspace is moderately (and nominally significantly) correlated with phylogenetic distance among species. Although this aspect of our work is somewhat exploratory and limited by available species sample size, we view this as preliminary evidence for phylogenetic signal in the shape of **ID**. Conversely, phylogenetic relatedness was not correlated with similarity in total among-individual variance (i.e., trace of **ID**) or alignment of **ID**_**max**_, suggesting that processes other than shared evolutionary history are more important in shaping these. Below we discuss our specific findings further, but with particular emphasis on the methodologies employed. In doing this, we hope to highlight several ways in which comparative, multivariate approaches might benefit the field of personality research.

### Total amount of among-individual variance in behavior

Although there is enormous empirical interest in testing hypotheses for the maintenance of personality variation within populations, very few studies have formally tested if populations (or species) differ in levels of among-individual variance (but see e.g., meta-analytic evidence for large-scale taxa differences in Bell et al. 2009). Here we show how comparison of **ID** matrices can be used to do this, and find that both *P. reticulata* and *D. rerio* exhibit significantly more variance than the other species. This is mainly driven by higher among-individual variance in Tl and TIM for these species. We currently have no specific biological hypothesis for this result. These two species were assayed in the smaller OFT arena and we cannot rule out the possibility that methodological differences in the assay are important. However, the smaller tank was used to reduce among-species differences in arena size relative to average body size. In fact, *P. reticulata* had the largest tank size to average body size ratio of all species (shown in Supplementary Table 1), although it was actually very similar to the ratios for *X. birchmanni* and *X. maculatus*. A strategy for resolving the potential influence of arena size would be to trial fish from a single species across a range of arena sizes to ask whether **ID** structure changes with environment (which actually implies plasticity of multivariate behavioral response to the OFT; Houslay and Wilson 2017). More generally, this approach—applied to multiple species and multiple environments simultaneously—could usefully allow assessment of whether among-species differences in levels of personality variation are themselves stable across defined environmental contexts.

### Interpretation of **ID**_max_ across species

As noted above, in only three (*X. birchmanni, L. nigrofasciata,* and *X. maculatus*) of the seven species did the trait loadings on **ID**_**max**_ correspond qualitatively to simple a priori expectations of a “shy-bold” axis. In two others (*X. hellerii* and *P. reticulata*), we can interpret **ID**_**max**_ as an axis of variance related to differences in flight-type stress response. In the remaining two species (*D. rerio* and *X. eiseni*) it is difficult to assign the leading eigenvector with any intuitive verbal label. Somewhat speculatively, in *X. eiseni* at least, this could be a consequence of pooling the sexes for analysis. Given available sample sizes, sex-specific analysis yield imprecise estimates (results not shown), but while the estimate of **ID**_**max**_ in male in *X. eiseni* appears consistent with a simple shy-bold axis, in females, it follows the putatively stress-related pattern.

The lack of a simple shy-bold axis in some species aligns with increasing reports that traits used as proxies for the same underlying personality trait can be uncorrelated. For instance, Beckmann and Biro 2013 found that two putative measures of boldness - emergence time in a novel environment and emergence time after a simulated predator attack are uncorrelated in damselfish (*Pomacentrus wardi* and *P. amboinsensis*). Similar results have been reported in studies of chacma baboons (*Papio ursinus*, Carter et al. 2012) and great tits (*Parus major*Arvidsson et al. 2017) and raises doubts about the extent to which univariate studies of “boldness” (or other personality traits) can be assumed to be targeting the same biological phenomenon. The fact that **ID**_**max**_ differs among even closely related species under standardized assays adds to this concern. There are two interpretations—either the OFT is not an appropriate tool for assaying boldness variation in all species tested, or the simple verbal model of a shy-bold continuum is not applicable to all fish species tested. Neither detracts in any way from the importance of testing hypotheses about among-individual variation, which is, of course, a prerequisite for adaptive evolution of behavior. However, both possibilities mean that attempts to generalize conclusions about boldness (or other personality traits) from the multitude of published single-species, single-proxy studies must proceed cautiously.

### Among-species similarity in higher dimensional subspace

In most species, **ID**_**max**_ represented just over half of the among-individual variation in **ID**, with the second eigenvector capturing the vast majority of the remainder. Furthermore, despite the fact that the species looked very different to each other when we sought to identify boldness from the first eigenvector, in two-dimensional subspace the behavioral variation was actually remarkably similar. It is tempting to conclude from this that the OFT reveals variation attributable to two latent personality traits. In some species, **ID**_**max**_ looks like a shy-bold axis while the second vector represents behavioral stress response. In others, the axes are structurally similar, but their relative importance is reversed.

Eigenvectors are mathematical properties of the (estimated) (co)variance matrix and are necessarily orthogonal to each other. Thus, for instance, the direction of the second eigenvector is not fully independent of the first. Consequently, where there are clear a priori hypotheses about the number of latent personality traits, and/or competing causal models for covariance among observed traits, it may generally be better to use approaches such as factor analysis (Araya-Ajoy and Dingemanse 2014), structural equation modeling (Dingemanse et al. 2010) or the recently suggested “EGA+GNM” framework (Martin et al. 2019) in conjunction with estimation of **ID**.

The current goal was really to ask if and how **ID** matrices differ among species, not to test competing biological hypotheses about why they differ. For the former, we think it sensible to describe (dis)similarity of multivariate variation using quantitative approaches designed for the task (rather than the imprecise language of personality traits). Here we have used the Krzanowski similarity index, a tool quite widely used in comparative quantitative genetics (Hine and Blows 2006; Teplitsky et al. 2013; Aguirre et al. 2014; Puentes et al. 2016), but other useful strategies are available (e.g., the Flury hierarchy; Phillips and Arnold 1999).

### Phylogenetic signal in among-individual variance in behavior

Finally, in asking whether phylogeny predicted not (multivariate) mean behavior, but rather the structure of among-individual variance itself, we found somewhat mixed results. Phylogenetic relatedness explained little of the among-species variation in **ID** trace, and effectively none of the variation in orientation of **ID**_**max**_. However, a moderate positive correlation was found between (estimated) phylogenetic distance and (estimated) 2-dimensional subspace dissimilarity. If such a pattern proved general in standardized testing, it could suggest that lower order aspects of **ID** covariance matrices (e.g., trace, direction of leading eigenvector) are more easily altered by selection (and/or drift) than higher order matrix “shape”. However, a counterexample is provided by subspace comparisons of phenotypic covariance structure among mating call traits in field crickets (Blankers et al. 2016), where no evidence for phylogenetic signal was detected in a comparison among seven species.

We fully acknowledge that seven species is not a particularly robust sample size from which to generalize the strength of phylogenetic signal in **ID**. Nonetheless, our finding is at least consistent with the idea that patterns of personality variation can display phylogenetic inertia. If so, then comparative studies of personality seeking to determine, for instance, what ecological factors might maintain high levels of variation within a population will need to control comparisons with respect to phylogeny. Though this need applies to any comparative analysis (Hansen and Orzack 2005), the pervasive view that behavior is more “evolutionarily labile” than other trait types (e.g., morphology, physiology) has perhaps led to an expectation that phylogenetic signals will be weak (Blomberg et al. 2003; Garamszegi et al. 2013). We suggest that more empirical studies would be valuable before accepting this general premise for behavioral averages, let alone for patterns of among-individual (co)variance. For the latter, a particular challenge arises in that standard phylogenetic methods are designed for modeling species differences in trait averages, not covariance matrices. Although the best approach is therefore not clear (at least to us), strategies for assessing stability of **G** matrices in quantitative genetic studies (Arnold et al. 2008) may be broadly applicable.

## CONCLUSION

The OFT does not reveal a simple dominant axis of “shy-bold” type variation that is consistent across the seven fish species tested. Rather species differ in both the total amount of among-individual variance, and in the orientation—and so biological interpretation—of the main axis of variation. This highlights exactly why it is presently difficult to compare inferences about “boldness” (or other latent personality traits) across studies. Nonetheless, by observing multiple traits in standardized tests and applying fully multivariate analyses, the structure of behavioral variation can be quantitatively compared with less reliance on ambiguous verbal labels. Here we find that, despite the major differences in the leading axes, 1) the “shape” of behavioral variation is actually rather similar across species when compared in more (two) dimensions; and 2) differences in shape that are present are predicted by phylogeny. We suggest that the field of animal personality research may have much to gain from greater uptake of multivariate analysis, and that combining this with comparative studies may be a valuable strategy to test evolutionary hypotheses.

## FUNDING

This work was supported by the Biotechnology and Biological Sciences Research Council (grant BB/L022656/1) and by a Natural Environment Research Council to S.J.W.

## Supplementary Material

arz198_suppl_Supplemental_materials

## Notes

Data accessibility: Analyses reported in this article can be reproduced using the data provided by White (2019).
