# Supplementary material for: Towards a comparative approach to the structure of animal personality variation
Source: Behav Ecol. 2019 Nov 26;31(2):340–51. doi: 10.1093/beheco/arz198 (PMC7083098; doi:10.1093/beheco/arz198)
Supplement: arz198_suppl_Supplemental_materials [file arz198_suppl_supplemental_materials.docx]

Supplemental table 1: Details of the data collection procedure on all 7 species. Includes the total number of individuals used, number of repeat measures of OFT, the size of the trial tank and the time period over which the repeat trials were performed. Tank size in fish body lengths was calculated using the species average standard length in each case.

| Species | Number of individuals | Maximum number of repeats | Experimental period | Maximum group size | Trial tank size (cm) | Trial tank size (fish body lengths) |
| --- | --- | --- | --- | --- | --- | --- |
| *D. rerio* | 26 | 6 | 5 weeks | 4 | 30 x 20 x20 | 9.43 x 6.29 x 6.29 |
| *L. nigrofasciata* | 32 | 4 | 2 weeks | 8 | 45 x 25 x 25 | 9.34 x 5.19 x 5.19 |
| *P. reticulata* | 831 | 4 | 2 weeks | 16 | 30 x 20 x20 | 15.41 x 10.27 x 10.27 |
| *X.birchmanni* | 369 | 9 | 28 weeks | 8 | 45 x 25 x 25 | 12.31 x 6.84 x 6.84 |
| *X. eiseni* | 36 | 4 | 2 weeks | 8 | 45 x 25 x 25 | 9.33 x 5.18 x 5.18 |
| *X. helleri* | 78 | 4 | 2 weeks | 78 | 45 x 25 x 25 | 9.52 x 5.29 x 5.29 |
| *X. maculatus* | 107 | 4 | 2 weeks | 18 | 45 x 25 x 25 | 14.79 x 8.22 x 8.22 |

Table 2: Mean and standard deviation values for each trait within each species on: the globally SD scaled data, the raw data (TIM no square root transformed but TL still in body length units) and TL in cm units.

| Species | Trait | Mean  (scaled globally) | SD  (scaled globally) | Mean  (raw data) | SD  (raw data) | Mean  TL in cm | SD  TL in cm |
| --- | --- | --- | --- | --- | --- | --- | --- |
| X. birchmanni | TL | -0.098 | 0.737 | 47.270 | 20.25 | 1728 | 740.4 |
|  | Act | 0.972 | 2.035 | 72.140 | 18.40 | - | - |
|  | AC | 0.104 | 0.767 | 39.590 | 12.37 | - | - |
|  | TIM | -0.466 | 0.686 | 27.850 | 30.84 | - | - |
| X.helleri | TL | -1.154 | 0.308 | 18.250 | 8.458 | 862.900 | 399.800 |
|  | Act | -0.809 | 0.632 | 27.140 | 15.96 | - | - |
|  | AC | -1.242 | 0.587 | 17.890 | 9.465 | - | - |
|  | TIM | -0.397 | 1.053 | 39.900 | 56.73 | - | - |
| P.reticulata | TL | 0.395 | 0.928 | 60.820 | 25.52 | 1184 | 496.800 |
|  | Act | -0.280 | 0.702 | 40.520 | 17.730 | - | - |
|  | AC | 0.037 | 0.942 | 38.510 | 15.180 | - | - |
|  | TIM | 0.252 | 1.013 | 69.280 | 62.860 | - | - |
| X.eiseni | TL | -0.975 | 0.302 | 23.16 | 8.306 | 1118 | 400.800 |
|  | Act | -0.339 | 0.682 | 39.020 | 17.410 | - | - |
|  | AC | 0.241 | 0.697 | 41.810 | 11.230 | - | - |
|  | TIM | -0.168 | 0.709 | 40.120 | 36.330 | - | - |
| L.nigrofasciata | TL | -1.229 | 0.191 | 16.190 | 5.242 | 779.700 | 252.500 |
|  | Act | -0.891 | 0.421 | 25.080 | 10.630 | - | - |
|  | AC | -0.103 | 0.692 | 36.250 | 11.190 | - | - |
|  | TIM | 0.091 | 0.656 | 51.510 | 37.570 | - | - |
| X.maculatus | TL | -1.016 | 0.444 | 22.030 | 12.210 | 670.400 | 371.5 |
|  | Act | -0.107 | 0.664 | 20.46 | 16.770 | - | - |
|  | AC | -0.441 | 0.960 | 30.800 | 15.480 | - | - |
|  | TIM | -0.155 | 1.036 | 49.300 | 56.180 | - | - |
| D.rerio | TL | 1.188 | 1.246 | 82.620 | 34.250 | 2629 | 1090 |
|  | Act | 1.492 | 0.785 | 85.280 | 19.820 | - | - |
|  | AC | 2.081 | 1.143 | 71.470 | 18.440 | - | - |
|  | TIM | 1.349 | 0.873 | 145.700 | 72.240 | - | - |

Supplemental table 3: sequence identifications and references used in the construction of the species relatedness matrix.

| Sequence ID | Species | Gene | Origin | Reference / DOI |
| --- | --- | --- | --- | --- |
| LC153764.1 | Limia nigrofasciata | COI | Japan: Aichi, Higashiyama Zoo | Unpublished |
| JN028266.1 | Poecilia reticulata | COI | Unknown | 10.1073/pnas.1016437108 |
| JN028265.1 | Poecilia reticulata | COI | Unknown | 10.1073/pnas.1016437108 |
| JQ432022.1 | Poecilia reticulata | COI | French Polynesia: Society Islands, Moorea, Criobe | 10.1371/journal.pone.0028987 |
| JN989141.1 | Poecilia reticulata | COI | Brazil: Distrito Federal, Upper Parana Basin | 10.1186/1471-2156-14-20 |
| JN989140.1 | Poecilia reticulata | COI | Brazil: Distrito Federal, Upper Parana Basin | 10.1186/1471-2156-14-20 |
| JN989139.1 | Poecilia reticulata | COI | Brazil: Distrito Federal, Upper Parana Basin | 10.1186/1471-2156-14-20 |
| JN989142.1 | Poecilia reticulata | COI | Brazil: Sao Paulo, Upper Parana Basin | 10.1186/1471-2156-14-20 |
| KM287034.1 | Poecilia reticulata | COI | Germany | 10.1111/1755-0998.12322 |
| KM287033.1 | Poecilia reticulata | COI | Germany | 10.1111/1755-0998.12322 |
| KM287032.1 | Poecilia reticulata | COI | Germany | 10.1111/1755-0998.12322 |
| KM287031.1 | Poecilia reticulata | COI | Germany | 10.1111/1755-0998.12322 |
| KT599873.1 | Poecilia reticulata | COI | Panama | 10.1371/journal.pone.0148040 |
| KT599872.1 | Poecilia reticulata | COI | Panama | 10.1371/journal.pone.0148040 |
| KT599871.1 | Poecilia reticulata | COI | Panama | 10.1371/journal.pone.0148040 |
| KT599870.1 | Poecilia reticulata | COI | Panama | 10.1371/journal.pone.0148040 |
| KT599869.1 | Poecilia reticulata | COI | Panama | 10.1371/journal.pone.0148040 |
| KT599868.1 | Poecilia reticulata | COI | Panama | 10.1371/journal.pone.0148040 |
| KT599867.1 | Poecilia reticulata | COI | Panama | 10.1371/journal.pone.0148040 |
| KT599866.1 | Poecilia reticulata | COI | Panama | 10.1371/journal.pone.0148040 |
| KT599865.1 | Poecilia reticulata | COI | Panama | 10.1371/journal.pone.0148040 |
| KT599864.1 | Poecilia reticulata | COI | Panama | 10.1371/journal.pone.0148040 |
| KT599863.1 | Poecilia reticulata | COI | Panama | 10.1371/journal.pone.0148040 |
| KT599862.1 | Poecilia reticulata | COI | Panama | 10.1371/journal.pone.0148040 |
| KU692776.1 | Poecilia reticulata | COI | Indonesia: Jawa Barat, West Java, Kabupaten Sukabumi, Danau Lido | 10.1111/1755-0998.12528 |
| KU692775.1 | Poecilia reticulata | COI | Indonesia: Jawa Barat, West Java, Kabupaten Sukabumi, Danau Lido | 10.1111/1755-0998.12528 |
| KU692774.1 | Poecilia reticulata | COI | Indonesia: Jawa Barat, West Java, Kabupaten Sukabumi, Danau Lido | 10.1111/1755-0998.12528 |
| KJ669589.1 | Poecilia reticulata | COI | Australia | Unpublished |
| GU702171.1 | Poecilia reticulata | COI | Brazil: Sao Paulo, Paraiba do Sul Basin | Unpublished |
| GU702170.1 | Poecilia reticulata | COI | Brazil: Sao Paulo, Paraiba do Sul Basin | Unpublished |
| GU702153.1 | Poecilia reticulata | COI | Brazil: Sao Paulo, Paraiba do Sul Basin | Unpublished |
| GU702152.1 | Poecilia reticulata | COI | Brazil: Sao Paulo, Paraiba do Sul Basin | Unpublished |
| GU702150.1 | Poecilia reticulata | COI | Brazil: Sao Paulo, Paraiba do Sul Basin | Unpublished |
| JQ734533.1 | Poecilia reticulata | COI | Cape Verde | Unpublished |
| JQ667563.1 | Poecilia reticulata | COI | India: Maharashtra, Mumbai, Dhobi Talao, Crawford Market | Unpublished |
| JQ667562.1 | Poecilia reticulata | COI | India: Maharashtra, Mumbai, Dhobi Talao, Crawford Market | Unpublished |
| KU568971.1 | Poecilia reticulata | COI | KwaZulu-Natal, Durban, South Africa | Unpublished |
| KU568970.1 | Poecilia reticulata | COI | KwaZulu-Natal, Durban, South Africa | Unpublished |
| MF591705.1 | Poecilia reticulata | COI | Western Ghats, Kerala, India | Unpublished |
| KJ844758.1 | Poecilia reticulata | COI | Aquarium sources | Vertebr Zool 65, 37-44 (2015) |
| AY356587.1 | Xenotoca eiseni | COI | Unknown, aquarium sources | 10.1016/S1055-7903(03) 00257-4 |
| JQ935946.1 | Xiphophorus birchmanni | COI | Mexico: Hidalgo, Huejutla de Reyes, Vado a 2 km al SO de Coacuilco | 10.3109/19401736.2012.710207 |
| JQ935944.1 | Xiphophorus birchmanni | COI | Mexico: Hidalgo, Huejutla de Reyes, Vado a 2 km al SO de Coacuilco | 10.3109/19401736.2012.710207 |
| JQ935945.1 | Xiphophorus birchmanni | COI | Mexico: Hidalgo, Huejutla de Reyes, Vado a 2 km al SO de Coacuilco | 10.3109/19401736.2012.710207 |
| EU752045.1 | Xiphophorus hellerii | COI | Guatemala: Alta Verapaz | 10.1111/j.1095-8649.2008.02077.x |
| EU752044.1 | Xiphophorus hellerii | COI | Guatemala: Alta Verapaz | 10.1111/j.1095-8649.2008.02077.x |
| EU752043.1 | Xiphophorus hellerii | COI | Guatemala: Alta Verapaz | 10.1111/j.1095-8649.2008.02077.x |
| EU752042.1 | Xiphophorus hellerii | COI | Guatemala: Alta Verapaz | 10.1111/j.1095-8649.2008.02077.x |
| EU752041.1 | Xiphophorus hellerii | COI | Guatemala: Alta Verapaz | 10.1111/j.1095-8649.2008.02077.x |
| EU752040.1 | Xiphophorus hellerii | COI | Guatemala: Alta Verapaz | 10.1111/j.1095-8649.2008.02077.x |
| EU752039.1 | Xiphophorus hellerii | COI | Guatemala: Alta Verapaz | 10.1111/j.1095-8649.2008.02077.x |
| EU752037.1 | Xiphophorus hellerii | COI | Guatemala: Alta Verapaz | 10.1111/j.1095-8649.2008.02077.x |
| EU752038.1 | Xiphophorus hellerii | COI | Guatemala: Alta Verapaz | 10.1111/j.1095-8649.2008.02077.x |
| KJ554749.1 | Xiphophorus hellerii | COI | Germany: Aquaculture drainage | 10.1111/1755-0998.12257 |
| KU692952.1 | Xiphophorus hellerii | COI | Indonesia: Bali, West Bali, Kab Buleleng, Danau Buyan | 10.1111/1755-0998.12528 |
| KU692950.1 | Xiphophorus hellerii | COI | Indonesia: Bali, West Bali, Kab Buleleng, Danau Buyan | 10.1111/1755-0998.12528 |
| KU692951.1 | Xiphophorus hellerii | COI | Indonesia: Jawa Barat, West Java, Kabupaten Purwakarta, Irrigation channel | 10.1111/1755-0998.12528 |
| KU692949.1 | Xiphophorus hellerii | COI | Indonesia: Jawa Barat, West Java, Kabupaten Purwakarta, Irrigation channel | 10.1111/1755-0998.12528 |
| KU692954.1 | Xiphophorus hellerii | COI | Indonesia: Jawa Barat, West Java, Kabupaten Sukabumi, S.Cisaat | 10.1111/1755-0998.12528 |
| KU692953.1 | Xiphophorus hellerii | COI | Indonesia: Jawa Barat, West Java, Kabupaten Sukabumi, S.Cisaat | 10.1111/1755-0998.12528 |
| KJ669651.1 | Xiphophorus hellerii | COI | Australia: Aquarium Trade | Hardy,C.M., 2014 CSIRO |
| HQ219151.1 | Xiphophorus hellerii | COI | India | 10.3109/19401736.2015.1101540 |
| HQ219150.1 | Xiphophorus hellerii | COI | India | 10.3109/19401736.2015.1101540 |
| HQ219148.1 | Xiphophorus hellerii | COI | India | 10.3109/19401736.2015.1101540 |
| HQ219147.1 | Xiphophorus hellerii | COI | India | 10.3109/19401736.2015.1101540 |
| HQ219149.1 | Xiphophorus hellerii | COI | India | 10.3109/19401736.2015.1101540 |
| JQ667588.1 | Xiphophorus hellerii | COI | India: Maharashtra, Mumbai, Dhobi Talao, Crawford Market | Unpublished |
| JQ667589.1 | Xiphophorus hellerii | COI | India: Maharashtra, Mumbai, Dhobi Talao, Crawford Market | Unpublished |
| HM345930.1 | Xiphophorus hellerii | COI | Indonesia | Unpublished |
| LC153769.1 | Xiphophorus hellerii | COI | Japan: Aichi, Higashiyama Zoo | Unpublished |
| KU569080.1 | Xiphophorus hellerii | COI | South Africa: Gauteng, Johannesburg, Rosetenville | - 10.2989/16085914.2017.1343178 |
| KU569076.1 | Xiphophorus hellerii | COI | South Africa: Gauteng, Johannesburg, Rosetenville | - 10.2989/16085914.2017.1343178 |
| KU569081.1 | Xiphophorus hellerii | COI | South Africa: KwaZulu-Natal, Durban | - 10.2989/16085914.2017.1343178 |
| KU569077.1 | Xiphophorus hellerii | COI | South Africa: KwaZulu-Natal, Durban | - 10.2989/16085914.2017.1343178 |
| KU569078.1 | Xiphophorus hellerii | COI | South Africa: Western Cape, Cape Town, Tyger Valley | - 10.2989/16085914.2017.1343178 |
| KU569079.1 | Xiphophorus hellerii | COI | South Africa: Western Cape, Cape Town, Tyger Valley | - 10.2989/16085914.2017.1343178 |
| KJ844768.1 | Xiphophorus hellerii | COI | Aquarium sources | Vertebr Zool 65, 37-44 (2015) |
| EU752052.1 | Xiphophorus maculatus | COI | Guatemala: Alta Verapaz | 10.1111/j.1095-8649.2008.02077.x |
| EU752051.1 | Xiphophorus maculatus | COI | Guatemala: Alta Verapaz | 10.1111/j.1095-8649.2008.02077.x |
| EU752050.1 | Xiphophorus maculatus | COI | Mexico: Quintana Roo | 10.1111/j.1095-8649.2008.02077.x |
| EU752049.1 | Xiphophorus maculatus | COI | Mexico: Quintana Roo | 10.1111/j.1095-8649.2008.02077.x |
| EU752048.1 | Xiphophorus maculatus | COI | Mexico: Quintana Roo | 10.1111/j.1095-8649.2008.02077.x |
| EU752047.1 | Xiphophorus maculatus | COI | Mexico: Quintana Roo | 10.1111/j.1095-8649.2008.02077.x |
| EU752046.1 | Xiphophorus maculatus | COI | Mexico: Quintana Roo | 10.1111/j.1095-8649.2008.02077.x |
| KU692957.1 | Xiphophorus maculatus | COI | Indonesia: Bali, West Bali, Kab Buleleng, Danau Buyan | 10.1111/1755-0998.12528 |
| KU692956.1 | Xiphophorus maculatus | COI | Indonesia: Bali, West Bali, Kab Buleleng, Danau Buyan | 10.1111/1755-0998.12528 |
| KU692955.1 | Xiphophorus maculatus | COI | Indonesia: Bali, West Bali, Kab Buleleng, Danau Buyan | 10.1111/1755-0998.12528 |
| KJ669652.1 | Xiphophorus maculatus | COI | Australia: Aquarium Trade | Hardy,C.M., 2014 CSIRO |
| JQ667593.1 | Xiphophorus maculatus | COI | India: Maharashtra, Mumbai, Dhobi Talao, Crawford Market | Unpublished |
| JQ667592.1 | Xiphophorus maculatus | COI | India: Maharashtra, Mumbai, Dhobi Talao, Crawford Market | Unpublished |
| JQ667591.1 | Xiphophorus maculatus | COI | India: Maharashtra, Mumbai, Dhobi Talao, Crawford Market | Unpublished |
| JQ667590.1 | Xiphophorus maculatus | COI | India: Maharashtra, Mumbai, Dhobi Talao, Crawford Market | Unpublished |
| KU569083.1 | Xiphophorus maculatus | COI | South Africa: KwaZulu-Natal, Durban | - 10.2989/16085914.2017.1343178 |
| KU569082.1 | Xiphophorus maculatus | COI | South Africa: KwaZulu-Natal, Durban | - 10.2989/16085914.2017.1343178 |
| KJ696814.1 | Limia nigrofasciata | Cytb | Unknown | 10.1038/nature13451 |
| U06488.1 | Poecilia reticulata | Cytb | Unknown | 10.1038/368539a0 |
| EF017536.1 | Poecilia reticulata | Cytb | Trinidad and Tobago: Aripo River | 10.1016/j.ympev.2006.06.009 |
| GU179192.1 | Poecilia reticulata | Cytb | Turure River, Trinidad | 10.1016/j.ympev.2009.11.006 |
| KJ013505.1 | Poecilia reticulata | Cytb | Unknown | 10.3109/19401736.2014.880902 |
| KJ415728.1 | Poecilia reticulata | Cytb | Venezuela | 10.1186/1471-2148-14-28 |
| KJ415723.1 | Poecilia reticulata | Cytb | Venezuela | 10.1186/1471-2148-14-28 |
| KJ415727.1 | Poecilia reticulata | Cytb | Venezuela | 10.1186/1471-2148-14-28 |
| KJ415720.1 | Poecilia reticulata | Cytb | Venezuela | 10.1186/1471-2148-14-28 |
| KJ415722.1 | Poecilia reticulata | Cytb | Venezuela | 10.1186/1471-2148-14-28 |
| KJ415724.1 | Poecilia reticulata | Cytb | Venezuela | 10.1186/1471-2148-14-28 |
| KJ415726.1 | Poecilia reticulata | Cytb | Venezuela | 10.1186/1471-2148-14-28 |
| KJ415705.1 | Poecilia reticulata | Cytb | Venezuela | 10.1186/1471-2148-14-28 |
| KJ415713.1 | Poecilia reticulata | Cytb | Venezuela | 10.1186/1471-2148-14-28 |
| KJ415707.1 | Poecilia reticulata | Cytb | Venezuela | 10.1186/1471-2148-14-28 |
| KJ415686.1 | Poecilia reticulata | Cytb | Venezuela | 10.1186/1471-2148-14-28 |
| KJ415683.1 | Poecilia reticulata | Cytb | Venezuela | 10.1186/1471-2148-14-28 |
| KJ415714.1 | Poecilia reticulata | Cytb | Venezuela | 10.1186/1471-2148-14-28 |
| KJ415712.1 | Poecilia reticulata | Cytb | Venezuela | 10.1186/1471-2148-14-28 |
| KJ415689.1 | Poecilia reticulata | Cytb | Venezuela | 10.1186/1471-2148-14-28 |
| KJ415706.1 | Poecilia reticulata | Cytb | Venezuela | 10.1186/1471-2148-14-28 |
| KJ415702.1 | Poecilia reticulata | Cytb | Venezuela | 10.1186/1471-2148-14-28 |
| KJ415704.1 | Poecilia reticulata | Cytb | Venezuela | 10.1186/1471-2148-14-28 |
| KJ415684.1 | Poecilia reticulata | Cytb | Venezuela | 10.1186/1471-2148-14-28 |
| KJ415690.1 | Poecilia reticulata | Cytb | Venezuela | 10.1186/1471-2148-14-28 |
| KJ415688.1 | Poecilia reticulata | Cytb | Venezuela | 10.1186/1471-2148-14-28 |
| KJ415682.1 | Poecilia reticulata | Cytb | Venezuela | 10.1186/1471-2148-14-28 |
| KJ415685.1 | Poecilia reticulata | Cytb | Venezuela | 10.1186/1471-2148-14-28 |
| KJ415700.1 | Poecilia reticulata | Cytb | Venezuela | 10.1186/1471-2148-14-28 |
| KJ415695.1 | Poecilia reticulata | Cytb | Venezuela | 10.1186/1471-2148-14-28 |
| KJ415698.1 | Poecilia reticulata | Cytb | Venezuela | 10.1186/1471-2148-14-28 |
| KJ415694.1 | Poecilia reticulata | Cytb | Venezuela | 10.1186/1471-2148-14-28 |
| KJ415699.1 | Poecilia reticulata | Cytb | Venezuela | 10.1186/1471-2148-14-28 |
| KJ415696.1 | Poecilia reticulata | Cytb | Venezuela | 10.1186/1471-2148-14-28 |
| KJ415692.1 | Poecilia reticulata | Cytb | Venezuela | 10.1186/1471-2148-14-28 |
| KJ415693.1 | Poecilia reticulata | Cytb | Venezuela | 10.1186/1471-2148-14-28 |
| KJ415697.1 | Poecilia reticulata | Cytb | Venezuela | 10.1186/1471-2148-14-28 |
| KJ415687.1 | Poecilia reticulata | Cytb | Venezuela | 10.1186/1471-2148-14-28 |
| KJ415715.1 | Poecilia reticulata | Cytb | Venezuela | 10.1186/1471-2148-14-28 |
| KJ415711.1 | Poecilia reticulata | Cytb | Venezuela | 10.1186/1471-2148-14-28 |
| KJ415710.1 | Poecilia reticulata | Cytb | Venezuela | 10.1186/1471-2148-14-28 |
| KJ415709.1 | Poecilia reticulata | Cytb | Venezuela | 10.1186/1471-2148-14-28 |
| KJ415708.1 | Poecilia reticulata | Cytb | Venezuela | 10.1186/1471-2148-14-28 |
| KJ415703.1 | Poecilia reticulata | Cytb | Venezuela | 10.1186/1471-2148-14-28 |
| KJ415725.1 | Poecilia reticulata | Cytb | Venezuela | 10.1186/1471-2148-14-28 |
| KJ415701.1 | Poecilia reticulata | Cytb | Venezuela | 10.1186/1471-2148-14-28 |
| KJ415691.1 | Poecilia reticulata | Cytb | Venezuela | 10.1186/1471-2148-14-28 |
| KJ415729.1 | Poecilia reticulata | Cytb | Venezuela | 10.1186/1471-2148-14-28 |
| KJ415679.1 | Poecilia reticulata | Cytb | Venezuela | 10.1186/1471-2148-14-28 |
| KJ415717.1 | Poecilia reticulata | Cytb | Venezuela | 10.1186/1471-2148-14-28 |
| KJ415721.1 | Poecilia reticulata | Cytb | Venezuela | 10.1186/1471-2148-14-28 |
| KJ415718.1 | Poecilia reticulata | Cytb | Venezuela | 10.1186/1471-2148-14-28 |
| KJ415719.1 | Poecilia reticulata | Cytb | Venezuela | 10.1186/1471-2148-14-28 |
| KJ415716.1 | Poecilia reticulata | Cytb | Venezuela | 10.1186/1471-2148-14-28 |
| KJ415681.1 | Poecilia reticulata | Cytb | Venezuela | 10.1186/1471-2148-14-28 |
| KJ415678.1 | Poecilia reticulata | Cytb | Venezuela | 10.1186/1471-2148-14-28 |
| KJ415680.1 | Poecilia reticulata | Cytb | Venezuela | 10.1186/1471-2148-14-28 |
| KP699838.1 | Poecilia reticulata | Cytb | Trinidad and Tobago: Guanapo River | 10.1371/journal.pone.0121139 |
| KP699837.1 | Poecilia reticulata | Cytb | Trinidad and Tobago: Guanapo River | 10.1371/journal.pone.0121139 |
| KP700413.1 | Poecilia reticulata | Cytb | Trinidad and Tobago: Trinidad | 10.1371/journal.pone.0121139 |
| KJ460033.1 | Poecilia reticulata | Cytb | Unknown | 10.1371/journal.pone.0169087 |
| GQ855708.1 | Poecilia reticulata | Cytb | Trinidad and Tobago: Isla de Margarita | Zootaxa 2266, 35-50 (2009) |
| GQ855721.1 | Poecilia reticulata | Cytb | Trinidad and Tobago: Trinidad, Caroni swamp | Zootaxa 2266, 35-50 (2009) |
| GQ855720.1 | Poecilia reticulata | Cytb | Trinidad and Tobago: Trinidad, Caroni swamp | Zootaxa 2266, 35-50 (2009) |
| GQ855736.1 | Poecilia reticulata | Cytb | Trinidad and Tobago: Trinidad, Claxton Bay | Zootaxa 2266, 35-50 (2009) |
| GQ855712.1 | Poecilia reticulata | Cytb | Trinidad and Tobago: Trinidad, Hillsborough | Zootaxa 2266, 35-50 (2009) |
| GQ855709.1 | Poecilia reticulata | Cytb | Trinidad and Tobago: Trinidad, Kaw | Zootaxa 2266, 35-50 (2009) |
| GQ855726.1 | Poecilia reticulata | Cytb | Trinidad and Tobago: Trinidad, Pitch lake | Zootaxa 2266, 35-50 (2009) |
| GQ855734.1 | Poecilia reticulata | Cytb | Trinidad and Tobago: Trinidad, Rio Guayre | Zootaxa 2266, 35-50 (2009) |
| GQ855710.1 | Poecilia reticulata | Cytb | Trinidad and Tobago: Trinidad, Rio Tefe | Zootaxa 2266, 35-50 (2009) |
| GQ855741.1 | Poecilia reticulata | Cytb | Trinidad and Tobago: Trinidad, Rio Yaguaracual | Zootaxa 2266, 35-50 (2009) |
| GQ855728.1 | Poecilia reticulata | Cytb | Trinidad and Tobago: Trinidad, Rio Yaguaracual | Zootaxa 2266, 35-50 (2009) |
| GQ855711.1 | Poecilia reticulata | Cytb | Trinidad and Tobago: Trinidad, Salimaos | Zootaxa 2266, 35-50 (2009) |
| GQ855735.1 | Poecilia reticulata | Cytb | Trinidad and Tobago: Trinidad, St. Annes | Zootaxa 2266, 35-50 (2009) |
| GQ855727.1 | Poecilia reticulata | Cytb | Trinidad and Tobago: Trinidad, upper Marianne | Zootaxa 2266, 35-50 (2009) |
| GQ855733.1 | Poecilia reticulata | Cytb | Aquarium trade | Zootaxa 2266, 35-50 (2009) |
| GQ855732.1 | Poecilia reticulata | Cytb | Aquarium trade | Zootaxa 2266, 35-50 (2009) |
| GQ855731.1 | Poecilia reticulata | Cytb | Aquarium trade | Zootaxa 2266, 35-50 (2009) |
| GQ855730.1 | Poecilia reticulata | Cytb | Aquarium trade | Zootaxa 2266, 35-50 (2009) |
| GQ855729.1 | Poecilia reticulata | Cytb | Aquarium trade | Zootaxa 2266, 35-50 (2009) |
| AF510766.1 | Xenotoca eiseni | Cytb | Mexico: Jalisco, Etzatlan | 10.1016/j.ympev.2003.08.022 |
| AF510765.1 | Xenotoca eiseni | Cytb | Mexico: Jalisco, Tamazula | 10.1016/j.ympev.2003.08.022 |
| AF510764.1 | Xenotoca eiseni | Cytb | Mexico: Jalisco, Tamazula | 10.1016/j.ympev.2003.08.022 |
| AP006777.1 | Xenotoca eiseni | Cytb | Unknown | 10.1016/j.ympev.2008.08.008 |
| KC778795.1 | Xenotoca eiseni | Cytb | Laboratorio de Biología Acuática, Universidad Michoacana de San Nicolás de Hidalgo | 10.1111/jfb.12611 |
| KP059018.1 | Xenotoca eiseni | Cytb | Mexico: El Moloya | 10.1643/CI-14-067 |
| KP059002.1 | Xenotoca eiseni | Cytb | Mexico: El Moloya | 10.1643/CI-14-067 |
| KP059003.1 | Xenotoca eiseni | Cytb | Mexico: El Moloya | 10.1643/CI-14-067) |
| KP059019.1 | Xenotoca eiseni | Cytb | Mexico: El Moloya | 10.1643/CI-14-067 |
| KP059007.1 | Xenotoca eiseni | Cytb | Mexico: Rio Compostela | 10.1643/CI-14-067 |
| KP059005.1 | Xenotoca eiseni | Cytb | Mexico: Rio Compostela | 10.1643/CI-14-067 |
| KP059004.1 | Xenotoca eiseni | Cytb | Mexico: Rio Compostela | 10.1643/CI-14-067 |
| KP058997.1 | Xenotoca eiseni | Cytb | Mexico: Rio Compostela | 10.1643/CI-14-067 |
| KP058996.1 | Xenotoca eiseni | Cytb | Mexico: Rio Compostela | 10.1643/CI-14-067 |
| KP059006.1 | Xenotoca eiseni | Cytb | Mexico: Rio Tamazula | 10.1643/CI-14-067 |
| KP059020.1 | Xenotoca eiseni | Cytb | Mexico: Rio Tamazula | 10.1643/CI-14-067 |
| KP059029.1 | Xenotoca eiseni | Cytb | Mexico: Rio Tamazula | 10.1643/CI-14-067 |
| KP059021.1 | Xenotoca eiseni | Cytb | Mexico: Rio Tamazula | 10.1643/CI-14-067 |
| KP059028.1 | Xenotoca eiseni | Cytb | Mexico: Rio Tamazula | 10.1643/CI-14-067 |
| KP059025.1 | Xenotoca eiseni | Cytb | Mexico: Rio Tamazula | 10.1643/CI-14-067 |
| KP059030.1 | Xenotoca eiseni | Cytb | Mexico: Rio Tamazula | 10.1643/CI-14-067 |
| KP059027.1 | Xenotoca eiseni | Cytb | Mexico: San Marcos | 10.1643/CI-14-067 |
| KP059026.1 | Xenotoca eiseni | Cytb | Mexico: San Marcos | 10.1643/CI-14-067 |
| KP059017.1 | Xenotoca eiseni | Cytb | Mexico: San Sebastian | 10.1643/CI-14-067 |
| KP059015.1 | Xenotoca eiseni | Cytb | Mexico: San Sebastian | 10.1643/CI-14-067 |
| KP059000.1 | Xenotoca eiseni | Cytb | Mexico: San Sebastian | 10.1643/CI-14-067 |
| KP059016.1 | Xenotoca eiseni | Cytb | Mexico: San Sebastian | 10.1643/CI-14-067 |
| KP059024.1 | Xenotoca eiseni | Cytb | Mexico: Seis de Enero | 10.1643/CI-14-067 |
| KP059023.1 | Xenotoca eiseni | Cytb | Mexico: Seis de Enero | 10.1643/CI-14-067 |
| KP059022.1 | Xenotoca eiseni | Cytb | Mexico: Seis de Enero | 10.1643/CI-14-067 |
| KP058999.1 | Xenotoca eiseni | Cytb | Mexico: Seis de Enero | 10.1643/CI-14-067 |
| KP059008.1 | Xenotoca eiseni | Cytb | Mexico: Seis de Enero | 10.1643/CI-14-067) |
| KP058998.1 | Xenotoca eiseni | Cytb | Mexico: Seis de Enero | 10.1643/CI-14-067 |
| KJ696850.1 | Xiphophorus birchmanni | Cytb | Unknown | 10.1038/nature13451 |
| EF017548.1 | Xiphophorus hellerii | Cytb | Unknown | 10.1016/j.ympev.2006.06.009 |
| FJ234985.1 | Xiphophorus hellerii | Cytb | Laboratory of Aquatic Laboratory Animals, Pearl River Fisheries Research Institute | 10.1080/19401730903033105 |
| JQ612909.1 | Xiphophorus hellerii | Cytb | Majahual River at Majahual, Los Tuxtlas | 10.1016/j.ympev.2012.09.010 |
| KJ696856.1 | Xiphophorus hellerii | Cytb | Unknown | 10.1038/nature13451 |
| AY056056.1 | Xiphophorus hellerii | Cytb | Unknown | Unpublished |
| U06511.1 | Xiphophorus maculatus | Cytb | Lago Catazaja | 10.1038/368539a0 |
| U06510.1 | Xiphophorus maculatus | Cytb | Rio Coatzacoalcos | 10.1038/368539a0 |
| U06514.1 | Xiphophorus maculatus | Cytb | Rio Jamapa | 10.1038/368539a0 |
| U06515.1 | Xiphophorus maculatus | Cytb | Rio Jamapa - east pop | 10.1038/368539a0 |
| U06512.1 | Xiphophorus maculatus | Cytb | Rio Papaloapan | 10.1038/368539a0 |
| U06513.1 | Xiphophorus maculatus | Cytb | Rio Usumacinta | 10.1038/368539a0 |
| EF017551.1 | Xiphophorus maculatus | Cytb | Unknown | 10.1016/j.ympev.2006.06.009 |
| AP005982.1 | Xiphophorus maculatus | Cytb | Unknown | 10.1098/rsos.150088 |
| DQ507808.1 | Xiphophorus maculatus | Cytb | Rio Jamapa | Zool. Abh. 55, 9-17 (2006) |
| KJ696906.1 | Limia nigrofasciata | ENC1 | Unknown | Unpublished |
| KJ696974.1 | Xenotoca eiseni | ENC1 | Unknown | Unpublished |
| KJ525873.1 | Xiphophorus birchmanni | ENC1 | Ryan Lab, University of Texas, Austin | 10.1111/evo.12391 |
| KJ525879.1 | Xiphophorus hellerii | ENC1 | Guatemala | 10.1111/evo.12391 |
| KJ697295.1 | Limia nigrofasciata | RAG1 | Unknown | Unpublished |
| EF017434.1 | Poecilia reticulata | RAG1 | Trinidad and Tobago: Aripo River | 10.1016/j.ympev.2006.06.009 |
| KJ697348.1 | Xenotoca eiseni | RAG1 | Unknown | Unpublished |
| KJ525813.1 | Xiphophorus birchmanni | RAG1 | Ryan Lab, University of Texas, Austin | 10.1111/evo.12391 |
| DQ235878.1 | Xiphophorus birchmanni | RAG1 | Unknown | Unpublished |
| EF017445.1 | Xiphophorus hellerii | RAG1 | Unknown | 10.1016/j.ympev.2006.06.009 |
| KJ525819.1 | Xiphophorus hellerii | RAG1 | Guatemala | 10.1111/evo.12391 |
| DQ235872.1 | Xiphophorus hellerii | RAG1 | Unknown | Unpublished |
| KJ844705.1 | Xiphophorus hellerii | RAG1 | Aquarium sources | Vertebr Zool 65, 37-44 (2015) |
| DQ235880.1 | Xiphophorus maculatus | RAG1 | Unknown | Unpublished |
| KX024091.1 | Limia nigrofasciata | Rhodopsin | Haiti: Etang de Miragoane | Unpublished |
| KX024090.1 | Limia nigrofasciata | Rhodopsin | Haiti: Etang de Miragoane | Unpublished |
| KJ697389.1 | Limia nigrofasciata | Rhodopsin | Unknown | Unpublished |
| AY141269.1 | Poecilia reticulata | Rhodopsin | Unknown | 10.1016/S1055-7903(02)00371-8 |
| DQ912023.1 | Poecilia reticulata | Rhodopsin | Trinidad and Tobago: Oropuche River, Trinidad | 10.1098/rspb.2006.3707 |
| DQ912024.1 | Poecilia reticulata | Rhodopsin | Trinidad and Tobago: Quare River, Trinidad | 10.1098/rspb.2006.3707 |
| GU179281.1 | Poecilia reticulata | Rhodopsin | Trinidad and Tobago: Turure River | 10.1016/j.ympev.2009.11.006 |
| NM_001312652.1 | Poecilia reticulata | Rhodopsin | Gabusoka River, Okinawa prefecture, Japan | 10.1186/1471-2148-11-81 |
| KJ697457.1 | Xenotoca eiseni | Rhodopsin | Unknown | Unpublished |
| KJ525793.1 | Xiphophorus birchmanni | Rhodopsin | Ryan Lab, University of Texas, Austin | 10.1111/evo.12391 |
| KJ525799.1 | Xiphophorus hellerii | Rhodopsin | Guatemala | 10.1111/evo.12391 |
| GU454735.1 | Xiphophorus hellerii | Rhodopsin | Rio Sarabia | Unpublished |
| KJ697499.1 | Limia nigrofasciata | SH3PX3 | Unknown | Unpublished |
| GU179224.1 | Poecilia reticulata | SH3PX3 | Trinidad and Tobago: Turure River | 10.1016/j.ympev.2009.11.006 |
| KJ697567.1 | Xenotoca eiseni | SH3PX3 | Unknown | Unpublished |
| KJ525773.1 | Xiphophorus birchmanni | SH3PX3 | Ryan Lab, University of Texas, Austin | 10.1111/evo.12391 |
| KJ525779.1 | Xiphophorus hellerii | SH3PX3 | Guatemala | 10.1111/evo.12391 |
| KJ697605.1 | Limia nigrofasciata | XSRC | Unknown | Unpublished |
| U06587.1 | Poecilia reticulata | XSRC | Unknown | 10.1038/368539a0 |
| DQ874651.1 | Poecilia reticulata | XSRC | Guyana: New Amsterdam | 10.1111/j.0014-3820.2006.tb01870.x |
| DQ874650.1 | Poecilia reticulata | XSRC | Guyana: New Amsterdam | 10.1111/j.0014-3820.2006.tb01870.x |
| DQ874649.1 | Poecilia reticulata | XSRC | Guyana: New Amsterdam | 10.1111/j.0014-3820.2006.tb01870.x10.x |
| DQ874654.1 | Poecilia reticulata | XSRC | Suriname: Lyledorp | 10.1111/j.0014-3820.2006.tb01870.x |
| DQ874653.1 | Poecilia reticulata | XSRC | Suriname: Lyledorp | 10.1111/j.0014-3820.2006.tb01870.x |
| DQ874652.1 | Poecilia reticulata | XSRC | Suriname: Lyledorp | 10.1111/j.0014-3820.2006.tb01870.x |
| DQ874670.1 | Poecilia reticulata | XSRC | Trinidad and Tobago: Arima River, west Trinidad | 10.1111/j.0014-3820.2006.tb01870.x |
| DQ874669.1 | Poecilia reticulata | XSRC | Trinidad and Tobago: Arima River, west Trinidad | 10.1111/j.0014-3820.2006.tb01870.x |
| DQ874668.1 | Poecilia reticulata | XSRC | Trinidad and Tobago: Arima River, west Trinidad | 10.1111/j.0014-3820.2006.tb01870.x |
| DQ874667.1 | Poecilia reticulata | XSRC | Trinidad and Tobago: Marianne River, west Trinidad | 10.1111/j.0014-3820.2006.tb01870.x |
| DQ874666.1 | Poecilia reticulata | XSRC | Trinidad and Tobago: Marianne River, west Trinidad | 10.1111/j.0014-3820.2006.tb01870.x |
| DQ874658.1 | Poecilia reticulata | XSRC | Trinidad and Tobago: Oropuche River, east Trinidad | 10.1111/j.0014-3820.2006.tb01870.x |
| DQ874657.1 | Poecilia reticulata | XSRC | Trinidad and Tobago: Oropuche River, east Trinidad | 10.1111/j.0014-3820.2006.tb01870.x |
| DQ874656.1 | Poecilia reticulata | XSRC | Trinidad and Tobago: Oropuche River, east Trinidad | 10.1111/j.0014-3820.2006.tb01870.x |
| DQ874655.1 | Poecilia reticulata | XSRC | Trinidad and Tobago: Oropuche River, east Trinidad | 10.1111/j.0014-3820.2006.tb01870.x |
| DQ874659.1 | Poecilia reticulata | XSRC | Trinidad and Tobago: Oropuche River,Trinidad | 10.1111/j.0014-3820.2006.tb01870.x |
| DQ874662.1 | Poecilia reticulata | XSRC | Trinidad and Tobago: Quare River, east Trinidad | 10.1111/j.0014-3820.2006.tb01870.x |
| DQ874661.1 | Poecilia reticulata | XSRC | Trinidad and Tobago: Quare River, east Trinidad | 10.1111/j.0014-3820.2006.tb01870.x |
| DQ874660.1 | Poecilia reticulata | XSRC | Trinidad and Tobago: Quare River, east Trinidad | 10.1111/j.0014-3820.2006.tb01870.x |
| DQ874665.1 | Poecilia reticulata | XSRC | Trinidad and Tobago: Yarra River, west Trinidad | 10.1111/j.0014-3820.2006.tb01870.x |
| DQ874664.1 | Poecilia reticulata | XSRC | Trinidad and Tobago: Yarra River, west Trinidad | 10.1111/j.0014-3820.2006.tb01870.x |
| DQ874663.1 | Poecilia reticulata | XSRC | Trinidad and Tobago: Yarra River, west Trinidad | 10.1111/j.0014-3820.2006.tb01870.x |
| DQ874635.1 | Poecilia reticulata | XSRC | Venezuela: Calle Caripe, Cumana | 10.1111/j.0014-3820.2006.tb01870.x |
| DQ874634.1 | Poecilia reticulata | XSRC | Venezuela: Calle Caripe, Cumana | 10.1111/j.0014-3820.2006.tb01870.x |
| DQ874638.1 | Poecilia reticulata | XSRC | Venezuela: Calle Margarita, Cumana | 10.1111/j.0014-3820.2006.tb01870.x |
| DQ874637.1 | Poecilia reticulata | XSRC | Venezuela: Calle Margarita, Cumana | 10.1111/j.0014-3820.2006.tb01870.x |
| DQ874636.1 | Poecilia reticulata | XSRC | Venezuela: Calle Margarita, Cumana | 10.1111/j.0014-3820.2006.tb01870.x |
| DQ874628.1 | Poecilia reticulata | XSRC | Venezuela: central Cumana | 10.1111/j.0014-3820.2006.tb01870.x |
| DQ874627.1 | Poecilia reticulata | XSRC | Venezuela: central Cumana | 10.1111/j.0014-3820.2006.tb01870.x |
| DQ874626.1 | Poecilia reticulata | XSRC | Venezuela: central Cumana | 10.1111/j.0014-3820.2006.tb01870.x |
| DQ874630.1 | Poecilia reticulata | XSRC | Venezuela: east Cumana | 10.1111/j.0014-3820.2006.tb01870.x |
| DQ874629.1 | Poecilia reticulata | XSRC | Venezuela: east Cumana | 10.1111/j.0014-3820.2006.tb01870.x |
| DQ874644.1 | Poecilia reticulata | XSRC | Venezuela: Mira Flores | 10.1111/j.0014-3820.2006.tb01870.x |
| DQ874643.1 | Poecilia reticulata | XSRC | Venezuela: Mira Flores | 10.1111/j.0014-3820.2006.tb01870.x |
| DQ874642.1 | Poecilia reticulata | XSRC | Venezuela: Mira Flores | 10.1111/j.0014-3820.2006.tb01870.x |
| DQ874645.1 | Poecilia reticulata | XSRC | Venezuela: Pozo Azufre | 10.1111/j.0014-3820.2006.tb01870.x |
| DQ874633.1 | Poecilia reticulata | XSRC | Venezuela: west Cumana | 10.1111/j.0014-3820.2006.tb01870.x |
| DQ874632.1 | Poecilia reticulata | XSRC | Venezuela: west Cumana | 10.1111/j.0014-3820.2006.tb01870.x |
| DQ874631.1 | Poecilia reticulata | XSRC | Venezuela: west Cumana | 10.1111/j.0014-3820.2006.tb01870.x |
| DQ874641.1 | Poecilia reticulata | XSRC | Venezuela: Yaguaracual | 10.1111/j.0014-3820.2006.tb01870.x |
| DQ874640.1 | Poecilia reticulata | XSRC | Venezuela: Yaguaracual | 10.1111/j.0014-3820.2006.tb01870.x |
| DQ874639.1 | Poecilia reticulata | XSRC | Venezuela: Yaguaracual | 10.1111/j.0014-3820.2006.tb01870.x |
| DQ874648.1 | Poecilia reticulata | XSRC | Venezuela: Yaguaraparo | 10.1111/j.0014-3820.2006.tb01870.x |
| DQ874647.1 | Poecilia reticulata | XSRC | Venezuela: Yaguaraparo | 10.1111/j.0014-3820.2006.tb01870.x |
| DQ874646.1 | Poecilia reticulata | XSRC | Venezuela: Yaguaraparo | 10.1111/j.0014-3820.2006.tb01870.x |
| GU179162.1 | Poecilia reticulata | XSRC | Turure River, Trinidad | 10.1016/j.ympev.2009.11.006 |
| KP700810.1 | Poecilia reticulata | XSRC | Trinidad and Tobago | 10.1371/journal.pone.0121139 |
| U02360.1 | Xenotoca eiseni | XSRC | Unknown | 10.1098/rspb.1993.0140 |
| KJ697673.1 | Xenotoca eiseni | XSRC | Unknown | Unpublished |
| U06596.1 | Xiphophorus birchmanni | XSRC | Rio Calabozo drainage | 10.1038/368539a0 |
| U06597.1 | Xiphophorus birchmanni | XSRC | Rio Chilcoaloya | 10.1038/368539a0 |
| KJ525893.1 | Xiphophorus birchmanni | XSRC | Ryan Lab, University of Texas, Austin | 10.1111/evo.12391 |
| U06608.1 | Xiphophorus hellerii | XSRC | Rio Coatzacoalcos | 10.1038/368539a0 |
| U06606.1 | Xiphophorus hellerii | XSRC | Rio Lancetilla | 10.1038/368539a0 |
| U06607.1 | Xiphophorus hellerii | XSRC | Rio Sarabia | 10.1038/368539a0 |
| KJ525899.1 | Xiphophorus hellerii | XSRC | Guatemala | 10.1111/evo.12391 |
| U06610.1 | Xiphophorus maculatus | XSRC | Lago Catazaja | 10.1038/368539a0 |
| U06609.1 | Xiphophorus maculatus | XSRC | Rio Coatzacoalcos | 10.1038/368539a0 |
| U06613.1 | Xiphophorus maculatus | XSRC | Rio Jamapa | 10.1038/368539a0 |
| U06611.1 | Xiphophorus maculatus | XSRC | Rio Papaloapan | 10.1038/368539a0 |
| U06612.1 | Xiphophorus maculatus | XSRC | Rio Usumacinta | 10.1038/368539a0 |
| U02362.1 | Xiphophorus maculatus | XSRC | Unknown | 10.1098/rspb.1993.0140 |
| U02361.1 | Xiphophorus maculatus | XSRC | Unknown | 10.1098/rspb.1993.0140 |

Supplemental table 4: Repeatabilities of OFT for 7 study species. Standard error in parentheses.

| Species | Trait | Repeatability V_I_/(V_I_+V_R_) | χ^2^_0,1_ | P |
| --- | --- | --- | --- | --- |
| *D. rerio* | Tl | 0.457 (0.097) | 40.32 | <0.001 |
|  | Act | 0.157 (0.084) | 5.49 | 0.009 |
|  | AC | 0.250 (0.091) | 14.58 | <0.001 |
|  | TIM | 0.562 (0.089) | 63.19 | <0.001 |
| L. nigrofasciata | Tl | 0.460 (0.095) | 30.32 | <0.001 |
|  | Act | 0.487 (0.093) | 34.13 | <0.001 |
|  | AC | 0.393 (0.097) | 22.29 | <0.001 |
|  | TIM | 0.312 (0.098) | 14.28 | <0.001 |
| *P. reticulata* | Tl | 0.543 (0.019) | 824.20 | <0.001 |
|  | Act | 0.473 (0.021) | 568.99 | <0.001 |
|  | AC | 0.403 (0.022) | 414.65 | <0.001 |
|  | TIM | 0.445 (0.021) | 531.32 | <0.001 |
| *X. birchmanni* | Tl | 0.225 (0.030) | 79.80 | <0.001 |
|  | Act | 0.266 (0.030) | 123.04 | <0.001 |
|  | AC | 0.166 (0.028) | 56.65 | <0.001 |
|  | TIM | 0.285 (0.030) | 135.16 | <0.001 |
| *X. eiseni* | Tl | 0.446 (0.090) | 31.47 | <0.001 |
|  | Act | 0.422 (0.092) | 27.94 | <0.001 |
|  | AC | 0.247 (0.092) | 10.14 | 0.001 |
|  | TIM | 0.385 (0.093) | 22.27 | <0.001 |
| *X. helleri* | Tl | 0.322 (0.063) | 36.55 | <0.001 |
|  | Act | 0.322 (0.063) | 36.61 | <0.001 |
|  | AC | 0.319 (0.063) | 35.00 | <0.001 |
|  | TIM | 0.246 (0.062) | 21.61 | <0.001 |
| *X. maculatus* | Tl | 0.346 (0.055) | 55.38 | <0.001 |
|  | Act | 0.393 (0.054) | 70.78 | <0.001 |
|  | AC | 0.326 (0.055) | 48.40 | <0.001 |
|  | TIM | 0.395 (0.054) | 69.42 | <0.001 |

Supplemental table 5: Test of significance of among-individual covariance structure of the 4 OFT traits in each of the 7 study species.

| Species | χ^2^_6_ | P |
| --- | --- | --- |
| *D. rerio* | 10.46 | 0.107 |
| L. nigrofasciata | 51.24 | <0.001 |
| *P. reticulata* | 820.48 | <0.001 |
| *X. birchmanni* | 147.79 | <0.001 |
| *X. eiseni* | 39.93 | <0.001 |
| *X. helleri* | 54.34 | <0.001 |
| *X. maculatus* | 103.44 | <0.001 |

Supplemental Table 6: Estimated **ID** matrices for the set of four OFT traits (Tl, Act, AC, TIM) for a) zebrafish (*Danio rerio*), b) Trinidadian guppy (*Poecilia reticulata*), c) red-tailed splitfin (*Xenotoca eiseni*), d) common platy (*Xiphophorus maculatus*), e) black-barred limia (*Lima nigrofasciata*), f) sheepshead swordtail (*Xiphophorus birchmanni*), and g) green swordtail (*Xiphophorus hellerii*). Among-individual variances are shown on the diagonal of each matrix (bold), with covariances (below the diagonal) also scaled to correlations (above diagonal). Standard errors are shown in parentheses. Note the diagonal elements are not equivalent to repeatabilities for each species (as in Supplemental Table 3) as data were converted to global (i.e. across all species) standard deviation units prior to analysis (see methods section of main text).

| (a) | *Tl* | *Act* | *AC* | *TIM* |  | (e) | *Tl* | *Act* | *AC* | *TIM* |
| --- | --- | --- | --- | --- | --- | --- | --- | --- | --- | --- |
| *Tl* | **0.462** (0.184) | 0.444  (0.281) | 0.672 (0.169) | -0.328 (0.230) |  | *Tl* | **0.016**  (0.005) | 0.996  (0.005) | 0.682 (0.141) | -0.264 (0.248) |
| *Act* | 0.079 (0.075) | **0.069**  (0.048) | 0.794 (0.170) | 0.002 (0.341) |  | *Act* | 0.036  (0.012) | **0.081**  (0.027) | 0.634 (0.150) | -0.285 (0.245) |
| *AC* | 0.234 (0.130) | 0.107  (0.068) | **0.262** (0.127) | -0.081 (0.283) |  | *AC* | 0.038  (0.016) | 0.080  (0.036) | **0.194**  (0.069) | 0.424  (0.227) |
| *TIM* | -0.143 (0.119) | <0.001  (0.057) | -0.027 (0.094) | **0.412** (0.138) |  | *TIM* | -0.012  (0.012) | -0.029  (0.028) | 0.067  (0.046) | **0.131**  (0.054) |
|  |  |  |  |  |  |  |  |  |  |  |
| (b) | *Tl* | *Act* | *AC* | *TIM* |  | (f) | *Tl* | *Act* | *AC* | *TIM* |
| *Tl* | **0.432**  (0.028) | 0.933 (0.006) | -0.191 (0.048) | -0.356 (0.041) |  | *Tl* | **0.106** (0.016) | 0.868  (0.027) | 0.561 (0.081) | 0.029 (0.104) |
| *Act* | 0.289  (0.020) | **0.222**  (0.016) | -0.139 (0.051) | -0.466 (0.038) |  | *Act* | 0.093 (0.015) | **0.109**  (0.016) | 0.573  (0.077) | 0.087  (0.101) |
| *AC* | -0.075  (0.019) | -0.039  (0.015) | **0.356**  (0.027) | 0.474 (0.040) |  | *AC* | 0.052  (0.013) | 0.054 (0.013) | **0.083** (0.016) | 0.705 (0.067) |
| *TIM* | -0.157  (0.023) | -0.148  (0.018) | 0.190  (0.023) | **0.453**  (0.032) |  | *TIM* | 0.003 (0.012) | 0.010  (0.012) | 0.069  (0.014) | **0.117**  (0.017) |
|  |  |  |  |  |  |  |  |  |  |  |
| (c) | *Tl* | *Act* | *AC* | *TIM* |  | (g) | *Tl* | *Act* | *AC* | *TIM* |
| *Tl* | **0.040**  (0.013) | 0.983 (0.008) | 0.650 (0.198) | -0.551 (0.182) |  | *Tl* | **0.030**  (0.008) | 0.988 (0.005) | 0.437 (0.152) | -0.396 (0.184) |
| *Act* | 0.085  (0.029) | **0.190**  (0.064) | 0.656 (0.202) | -0.615  (0.171) |  | *Act* | 0.061  (0.016) | **0.126**  (0.033) | 0.447 (0.151) | -0.454 (0.177) |
| *AC* | 0.043  (0.020) | 0.095 (0.045) | **0.109**  (0.050) | 0.120 (0.283) |  | *AC* | 0.024  (0.011) | 0.050  (0.023) | **0.099**  (0.027) | 0.388 (0.179) |
| *TIM* | -0.046  (0.022) | -0.112  (0.051) | 0.017  (0.041) | **0.174**  (0.063) |  | *TIM* | -0.034  (0.018) | -0.081  (0.037) | 0.061  (0.035) | **0.249**  (0.079) |
|  |  |  |  |  |  |  |  |  |  |  |
| (d) | *Tl* | *Act* | *AC* | *TIM* |  |  |  |  |  |  |
| *Tl* | **0.053**  (0.013) | 0.983 (0.007) | 0.918 (0.062) | 0.574 (0.161) |  |  |  |  |  |  |
| *Act* | 0.084  (0.020) | **0.136**  (0.030) | 0.915 (0.061) | 0.592 (0.151) |  |  |  |  |  |  |
| *AC* | 0.117  (0.026) | 0.187  (0.039) | **0.306**  (0.066) | 0.805 (0.087) |  |  |  |  |  |  |
| *TIM* | 0.076  (0.023) | 0.126  (0.035) | 0.257  (0.058) | **0.332**  (0.075) |  |  |  |  |  |  |

Supplemental table 7: Pairwise comparison of **ID** structure between each species pair, with χ^2^ at 10 DF. Species: X. birchmanni (Xb), X. helleri (Xh), X. maculatus (Xm), X. eiseni (Xe), L. nigrofasciata (Ln), P. reticulata (Pr) and D. rerio (Dr).

| Species paring | χ^2^ | P |
| --- | --- | --- |
| I_Xb_ - I_Xh_ | 64.98 | <0.001 |
| I_Xb_ - I_Xb_ | 69.94 | <0.001 |
| I_Xh_ - I_Xe_ | 5.41 | 0.862 |
| I_Xb_ - I_Ln_ | - | - |
| I_Xh_ - I_Ln_ | - | - |
| I_Xe_ - I_Ln_ | - | - |
| I_Xb_ - I_Xm_ | 79.26 | <0.001 |
| I_Xh_ - I_Xm_ | 40.28 | <0.001 |
| I_Xe_ - I_Xm_ | 42.91 | <0.001 |
| I_Ln_ - I_Xm_ | - | - |
| I_Xb_ - I_Pr_ | 556.48 | <0.001 |
| I_Xh_ - I_Pr_ | 345.40 | <0.001 |
| I_Xe_ - I_Pr_ | 172.08 | <0.001 |
| I_Ln_ - I_Pr_ | - | - |
| I_Xm_ - I_Pr_ | 388.44 | <0.001 |
| I_Xb_ - I_Dr_ | 77.12 | <0.001 |
| I_Xh_ - I_Dr_ | 72.80 | <0.001 |
| I_Xe_ - I_Dr_ | 79.42 | <0.001 |
| I_Ln_ - I_Dr_ | - | - |
| I_Xm_ - I_Dr_ | 71.70 | <0.001 |
| I_Pr_ - I_Dr_ | 67.8 | <0.001 |
